# Emergence of Motor Circuit Activity

**DOI:** 10.1371/journal.pone.0093836

**Published:** 2014-04-10

**Authors:** Chris Law, Michel Paquet, Artur Kania

**Affiliations:** 1 Institut de recherches cliniques de Montréal (IRCM), Montréal, Canada; 2 Departments of Anatomy and Cell Biology, and Biology, Division of Experimental Medicine, McGill University Montréal, Montréal, Canada, and Faculté de Médecine, Université de Montréal, Montréal, Canada; Emory University, United States of America

## Abstract

In the developing nervous system, ordered neuronal activity patterns can occur even in the absence of sensory input and to investigate how these arise, we have used the model system of the embryonic chicken spinal motor circuit, focusing on motor neurons of the lateral motor column (LMC). At the earliest stages of their molecular differentiation, we can detect differences between medial and lateral LMC neurons in terms of expression of neurotransmitter receptor subunits, including *CHRNA5*, *CHRNA7*, *GRIN2A*, *GRIK1*, *HTR1A* and *HTR1B*, as well as the KCC2 transporter. Using patch-clamp recordings we also demonstrate that medial and lateral LMC motor neurons have subtly different activity patterns that reflect the differential expression of neurotransmitter receptor subunits. Using a combination of patch-clamp recordings in single neurons and calcium-imaging of motor neuron populations, we demonstrate that inhibition of nicotinic, muscarinic or GABA-ergic activity, has profound effects of motor circuit activity during the initial stages of neuromuscular junction formation. Finally, by analysing the activity of large populations of motor neurons at different developmental stages, we show that the asynchronous, disordered neuronal activity that occurs at early stages of circuit formation develops into organised, synchronous activity evident at the stage of LMC neuron muscle innervation. In light of the considerable diversity of neurotransmitter receptor expression, activity patterns in the LMC are surprisingly similar between neuronal types, however the emergence of patterned activity, in conjunction with the differential expression of transmitter systems likely leads to the development of near-mature patterns of locomotor activity by perinatal ages.

## Introduction

Spontaneous neuronal activity, in the absence of sensory stimulation, is a hallmark of developing neuronal systems such as the cortex, hippocampus, retina, cerebellum and spinal cord [1]–[6]. The diverse neuronal populations that make up each of these systems are thought to interact with each other to shape the early disorganized activity patterns into more mature ones, but how this occurs is still a matter of debate. Spontaneously-arising activity is postulated to affect aspects of circuit formation in aquatic [7]–[9] and terrestrial organisms [5], [10], [11], though the extent of this activity-induced plasticity is under debate [12]. It is likely that, given the influence of activity upon synaptic plasticity, and the complex patterns of activity in perinatal circuits [1], [6], [13]–[15], the early patterns of neuronal activity and the mechanisms underlying them are critical to the normal development of mature activity patterns.

We chose to investigate the mechanisms underlying the maturation of activity patterns using the motor circuit of the chick spinal cord as a model system, the components of which have been well-studied, along with the molecular mechanisms resulting in their generation. The progenitors from which motor neurons arise have been identified [16]–[18], along with the molecular mechanisms controlling their differentiation into subpopulations such as columns and muscle-specific pools [17], [19], [20]. Motor neurons become electrically active very soon after their differentiation and these cholinergic neurons, alongside GABA-ergic interneurons are the source of rhythmic activity at early stages [4], [21], [22]; at later stages this cholinergic drive is replaced by glutamatergic neurons [23], demonstrating a degree of plasticity within the developing circuit. Ventral root recordings and calcium imaging from early chick embryos reveal that prior to the onset of sensory input [24] motor neurons fire ‘episodes’ of activity consisting of one or more large depolarisations (‘cycles’), followed by periods of depression [4], [25], [26]. This pattern of activity matures over time, such that in older embryos the period between episodes is significantly longer, and each episode contains more cycles [27]–[29]. Differences between the activity patterns of motor pools are critical for the normal function of the motor circuit; at perinatal ages, ipsilateral extensor and flexor motor pools fire alternating episodes of activity that direct the contraction of their respective muscles to generate a walking pattern, which is critically dependent on both motor neuron and interneuron electrical activity refinement.

Previous studies have investigated the contributions of neurotransmitter systems to spontaneous motor activity using pharmacological [23], [27], [30], [31] and genetic means [32]. Considerably fewer groups have examined the developmental dynamics of expression of neurotransmitter receptors and related proteins [33], leaving our understanding of the contribution of neurotransmitter systems to early circuit formation quite fragmented. Here, we examine the early stages of motor neuron development, focussing on neurotransmitter identity and receptor complement of neurons of the chick spinal motor circuit, and how these may influence differences in electrical characteristics and firing patterns of limb-innervating motor neurons. Furthermore, we examine how spinal neurotransmission shapes motor circuit function, by examining the effects of the loss of function of specific neurotransmitters on activities of individual motor neurons and their populations. Finally, we demonstrate and characterise the emergence of synchronous activity across the motor system during early spinal circuit development.

## Results

### Expression of Neurotransmitter Receptors in the Developing Motor Circuit

We hypothesized that the various patterns of electrical activity that occur in specific motor pools [28] at relatively late stages of chick embryonic development (embryonic day 12–14) may arise as a result of differential expression of neurotransmitters between subpopulations of neurons within the motor circuit at earlier stages. To investigate this question, we examined the expression of mRNAs encoding 36 neurotransmitter (NT) receptor subunits and other markers of NT identity in the lumbar spinal cord of the chick at the following stages: HH St. 24 (E4, “*early*”) at the end of the generation of lateral LMC neurons [34], HH St. 26 (E5, “*mid*”), when LMC axons are making a critical dorsoventral growth choice at the base of the limb [35] and HH St. 30 (E6, “*late*”), when LMC axons are innervating muscle targets in the limb [36]. To carry out this analysis, we combined *in situ* mRNA detection with immunofluorescence antibody detection in adjacent sections. The markers were pre-selected based on expression patterns found in the Genepaint (http://genepaint.org) and St. Judes (http://www.stjudebgem.org/; no longer active) expression databases in E14.5/E15 mouse embryos, when basic spinal locomotor circuits are present and already active [4], [36]. In addition to these markers of neurotransmitter identity, we performed *in situ* detection of *ISL1* and *LHX1* mRNAs, respective markers of the medial and lateral divisions of the Lateral Motor Column (LMC), to allow us to differentiate between the two cardinal divisions of limb-innervating motor neurons, innervating flexor and extensor limb muscles, respectively [20], [37].

**Figure 1:**
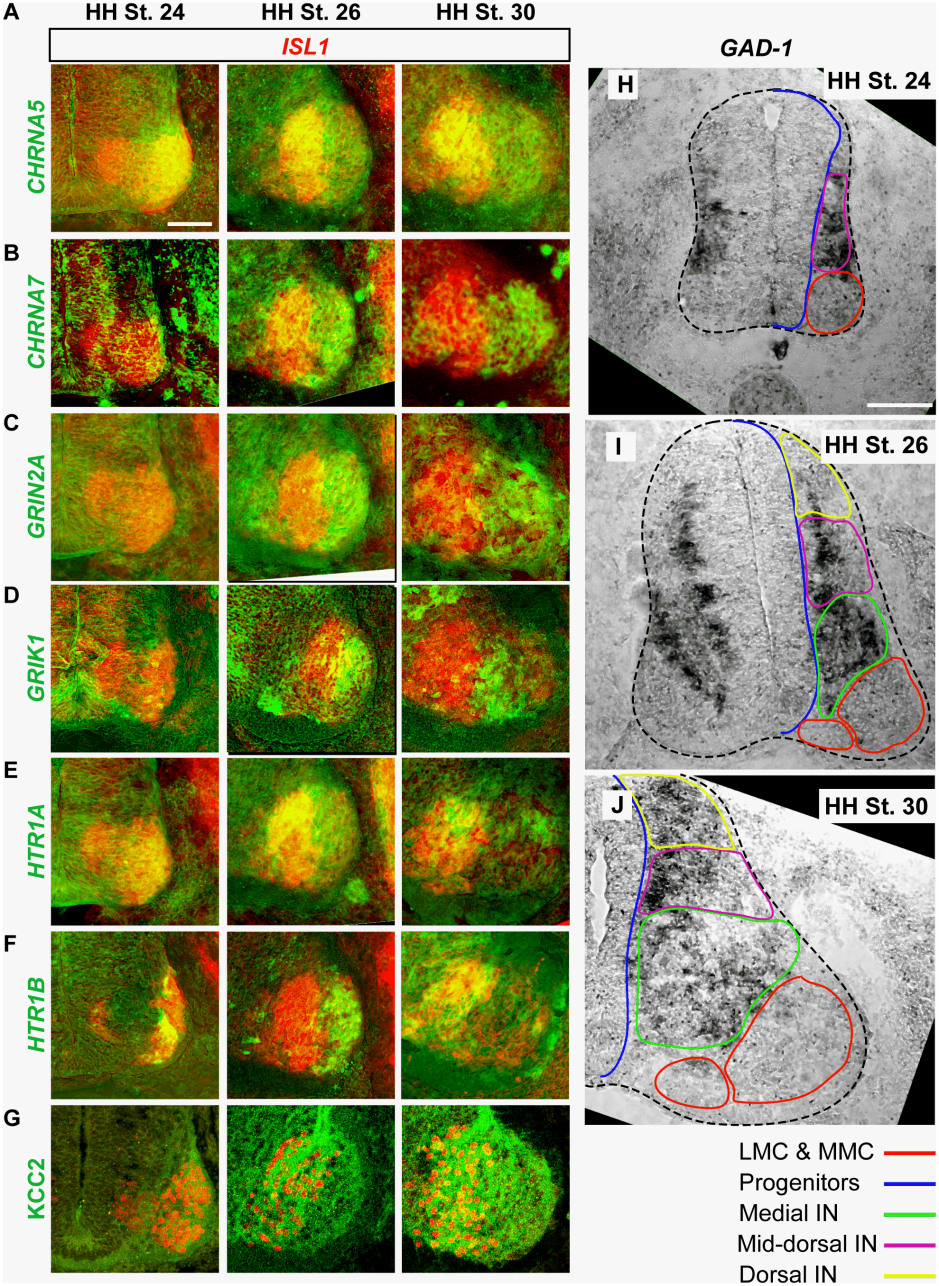
Expression of neurotransmitters and neurotransmitter receptors in the early motor circuit. A–H) In-situ detection of neurotransmitter receptor subunit mRNA (green) and *Isl1* mRNA (red) in adjacent sections reveals differential expression in medial LMC and lateral LMC motor neurons from HH St. 24–30. G) KCC2 (green) and Isl1 (red) protein detection. H–J) *GAD-1* mRNA expression in the whole spinal cord (dotted line) from HH St. 24–30. Coloured contours of spinal regions correspond to those summarizing neurotransmitter receptor expression in Table 1. Scale bars represent 100 μm.

Many mRNAs encoding NT receptor subunits were expressed throughout the ventral spinal cord from early to late stages, whereas some were preferentially expressed in interneurons located in the intermediate spinal cord, medial or dorsal to LMC motor neurons. Fig. 1 summarizes the selective patterns of gene expression within motor neurons, whereas Table 1 summarizes all gene expression patterns; unless a figure reference is given, all data can be found in Table 1. To describe the differential expression of markers of NT identity and NT receptors in interneuron populations, we defined boundaries based upon the pattern of expression relative to markers of the LMC (Fig. 1 H–J), and described expression patterns in terms of these boundaries. The boundary of the LMC was defined at *early* stages as being where the lateral extent of *LHX1* expression meets *ISL1* expression. At *mid* to *late* stages, the LMC border was defined by the medial extent of *ISL1* expression. The “medial interneuron” boundary was defined as the region encompassing the interneurons medial to the LMC, as well as those up to 100 μm dorsal to the LMC. At *early* stages this boundary does not exist, as only progenitors exist medial to the LMC. The “dorsal interneuron” boundary encompasses the dorsal-most quarter of the spinal cord, and the “mid-dorsal cord interneuron” boundary encompasses all other postmitotic neurons. The “progenitor” boundary is identified by cells that have mediolateral orientation. Our analysis does not take account for the presence of glial cells in the spinal cord. Using relative terms, expression patterns were described as “enriched” if there was increased expression in the given region when compared with an adjacent region (i.e. lateral LMC vs. medial LMC); or “specific” if only the region described showed any signal relative to the rest of the spinal cord. Where “no expression” is reported in the spinal cord, the quality of the probe was tested by signal detection in the spinal cord at later stages, or in other tissues.

**Table 1:** mRNA Enrichment in Spinal Neuron Populations.

Gene	HH St. 24	HH St. 26	HH St. 30
***GAD1***	Mid-dorsal cord IN	Medial, mid-dorsal cord and ventral IN	Medial, mid-dorsal cord and ventral IN
***GAD2***	Mid-dorsal cord IN specific	Medial, mid-dorsal cord and ventral IN	Medial, mid-dorsal cord and ventral IN
***GABRA3***	LMC, mid-dorsal cord IN	LMC, mid-dorsal cord IN	LMC, mid-dorsal cord IN
***GABRB3***	LMC, mid-dorsal cord IN	LMC, mid-dorsal cord IN	LMCm, mid-dorsal cord IN
***GABRG2***	Scattered through Cord	Scattered through Cord	Scattered through Cord
***GABBR2***	Scattered through Cord	Scattered through Cord	Scattered through Cord
***ChAT***	LMC-specific	LMC-specific	LMC-specific
***CHRM4***	LMC-specific	LMC-specific	LMC-specific
***CHRM5***	Weak in progenitors (specific)	Progenitor specific	Progenitor specific
***CHRNA2***	Pan-neuronal	Pan-neuronal	Pan-neuronal
***CHRNA3***	Pan-neuronal	Pan-neuronal	Pan-neuronal
***CHRNA5***	LMC specific	LMC (higher in LMCm)	LMC (higher in dorsal LMCm)
***CHRNA7***	No expression	LMC (higher in LMCl)	LMC (higher in LMCl)
***CHRNB2***	LMC specific	LMC specific	LMC specific
***SERT1***	No expression	No expression	No expression
***HTR1A***	Whole LMC	LMC (higher in dorsal LMCm)	LMC (higher in dorsal LMCm)
***HTR1B***	Few LMC neurons	LMCl	LMCm
***HTR4A***	Progenitors (weak), LMC	Weak Pan-LMC	Weak Pan-LMC
***GLYT2***	Interneurons	Interneurons	Interneurons
***GLRA1***	Pan-neuronal	Pan-neuronal	Pan-neuronal
***GLRA2***	LMC (weak)	Pan-LMC, some more dorsal IN	Pan-LMC, some more dorsal IN
***GLRB***	No Expression	Pan-LMC	Pan-LMC
***VGLUT2***	Interneurons	Interneurons	Interneurons
***GRIN1***	LMC (weak)	LMC, MMC, ventral IN, mid-dorsal cord IN	LMC, MMC, ventral IN, mid-dorsal cord IN
***GRIN2A***	No expression	LMCl, some dorsal IN	LMCl, medial IN, dorsal IN
***GRIK1***	Sparse ventromedial IN, dorsal IN	LMCl, sparse medial IN, dorsal IN, ventral progenitors	LMCl, sparse medial IN, ventral progenitors
***GRIK2***	Pan-LMC	Pan-LMC	Pan-LMC
***GRIA1***	Scattered IN	Dorsal IN, scattered ventral IN, specific progenitor domain	Dorsal IN, scattered ventral IN, specific progenitor domain, Scattered LMC
***GRIA2***	All postmitotic neurons	All postmitotic neurons	All postmitotic neurons
***GRM3***	Scattered progenitors	Progenitor-specific	Progenitor-specific
***GRM4***	Ventral LMC specific	Pan-neuronal	Pan-neuronal
***GRM5***	Weak in ventral LMC	Weak in LMC	Weak in LMC
***GRM7***	LMC (weak), medial Isl1^+^ progenitors	LMC (higher in LMCm),	LMC (higher in LMCm), dorsal IN, medial IN
***GRM8***	Progenitors, ventral LMC	Progenitors, scattered LMC neurons	Progenitors, scattered LMC neurons
***KCC2***	LMC (weak)	LMC (higher in LMCm)	LMC (higher in LMCm)
***NKCC1***	Weak expression on all LMC soma	Weak expression on all LMC soma	Weak expression on all LMC soma

This table describes the enrichment of mRNAs in spinal cord regions defined in the Results section at various stages.

As expected, Choline Acetyl Transferase (ChAT) is specific to motor neurons at all stages examined. mRNA from genes encoding some acetylcholine receptors (*CHRM4*, *CHRNA2*, *CHRNA3*, *CHRNB2*) is enriched in the LMC, or expressed throughout the spinal cord from *early* to *late* stages, whereas *CHRNA5* and *CHRNA7* are enriched in medial LMC neurons (early to late stages, Fig. 1A) and lateral LMC neurons (mid to late stages, Fig. 1B), respectively. The expression of *CHRM5* appears to be specific to the progenitor domains of the spinal cord, though it is expressed throughout the dorsoventral extent of this domain.

*GAD-1* (Fig. 1H) and *GAD-2*, two genes encoding enzymes responsible for the synthesis of GABA, are not expressed in LMC neurons, but are enriched in interneurons dorsal to the LMC at early stages, and dorsomedial to the LMC at mid to late stages (Fig. 1H–J). Of the genes encoding receptors for GABA, *GABRA3* is expressed throughout the LMC from *early* to *late* stages, whilst *GABRB3* is expressed in all LMC neurons from *early* to *late* stages, though its expression at *late* stages is enriched in medial LMC neurons when compared to lateral LMC neurons (Table 1). *GABBR2*, the gene encoding the sole GABA-B receptor subunit expressed in the spinal cord is detected, but its mRNA was only weakly expressed in neurons scattered throughout the spinal cord, as was *GABRG2* (Table 1).

*GLYT2* mRNA is expressed in a pattern similar to *GAD1* from *early* to *late* stages (Table 1), and several glycine receptors are expressed in the LMC or ventral spinal cord. *GLRA1* is expressed throughout the spinal cord, while *GLRA2* mRNA is enriched in both the whole LMC, and some interneurons dorsal to the LMC from *mid* to *late* stages (Table 1). *GLRB1* is specific to the LMC from *mid* to *late* stages.

*VGLUT2* expression is specific to interneurons in the more dorsal regions of the spinal cord at *early* stages; from *mid* to *late* stages, *VGLUT2* expression is also found in interneurons adjacent to the LMC. Of the genes encoding ionotropic glutamate receptors, *GRIN1* expression is enriched in the ventral spinal cord, particularly in the LMC, whilst *GRIN2A* is enriched in the lateral LMC from *mid* to *late* stages (Fig. 1C), as well as in interneurons dorsomedial to the LMC at *late* stages. *GRIK2* expression is enriched in LMC, whilst *GRIK1* is specific to interneurons ventromedial to the LMC from *early* to *late* stages, in addition to lateral LMC neurons from *mid* to *late* stages (Fig. 1D). *GRIA1* expression is enriched in mid-dorsal cord and dorsal interneurons and a specific group of progenitors at all stages examined; from *mid* to *late* stages, expression is also enriched in medial interneurons. *GRIA2* is expressed in all post-mitotic neurons. Of the genes encoding metabotropic glutamate receptors, *GRM3* is specific to progenitors from *mid* to *late* stages; *GRM4* expression is specific to the ventral LMC at *early* stages, but spreads to the entire spinal cord by mid stages; *GRM5* expression is enriched in the LMC, with highest expression in the medial LMC; *GRM7* is specific to the LMC, and enriched in the medial LMC. *GRM8* expression is enriched in progenitors at all stages examined, and high in scattered neurons throughout the spinal cord, though expression is also somewhat enriched in the LMC.

No *SERT-1* mRNA expression was detected in the spinal cord at any of the stages examined, though the probe was able to detect *SERT-1* in the hindbrain (data not shown). Conversely, mRNAs for multiple 5-HT receptors are expressed in the spinal cord. *HTR1A mRNA* is enriched in the dorsal LMC from *mid* to *late* stages (Fig. 1C), whereas *HTR1B* expression appears to switch from being enriched in the lateral LMC at *mid* stages to the medial LMC at *late* stages (Fig. 1D). *HTR4A* is enriched in the LMC from *early* to *late* stages, albeit at low levels.

Using immunocytochemistry, we also assayed the expression of the chloride transporters NKCC1 and KCC2, which regulate internal chloride concentration and thus the polarity of GABA-induced currents [38]. From *early* to *mid* stages, KCC2 is enriched in medial LMC motor neurons, though by *late* stages lateral LMC neurons also began to express KCC2, albeit at lower levels than in medial LMC neurons (Fig. 1G). In contrast, NKCC1 expression appears to be uniform throughout the LMC at the stages examined, with little expression found on motor neuron soma, but high levels on neuronal processes.

Together, these data demonstrate the dynamic expression patterns of the genes encoding neurotransmitter receptors, synthetic enzymes and transporters in spinal interneurons and motor neurons at the developmental stages examined, suggesting that cholinergic, GABA-ergic, glutamatergic, serotonergic and glycinergic neurotransmitter systems are competent to influence the very earliest motor circuit activity [4]. Furthermore, we found that motor neurons express a variety of neurotransmitter receptors, with some being differentially expressed between motor neuron subpopulations.

### Electrical Characteristics of LMCm and LMCl Neurons

**Figure 2:**
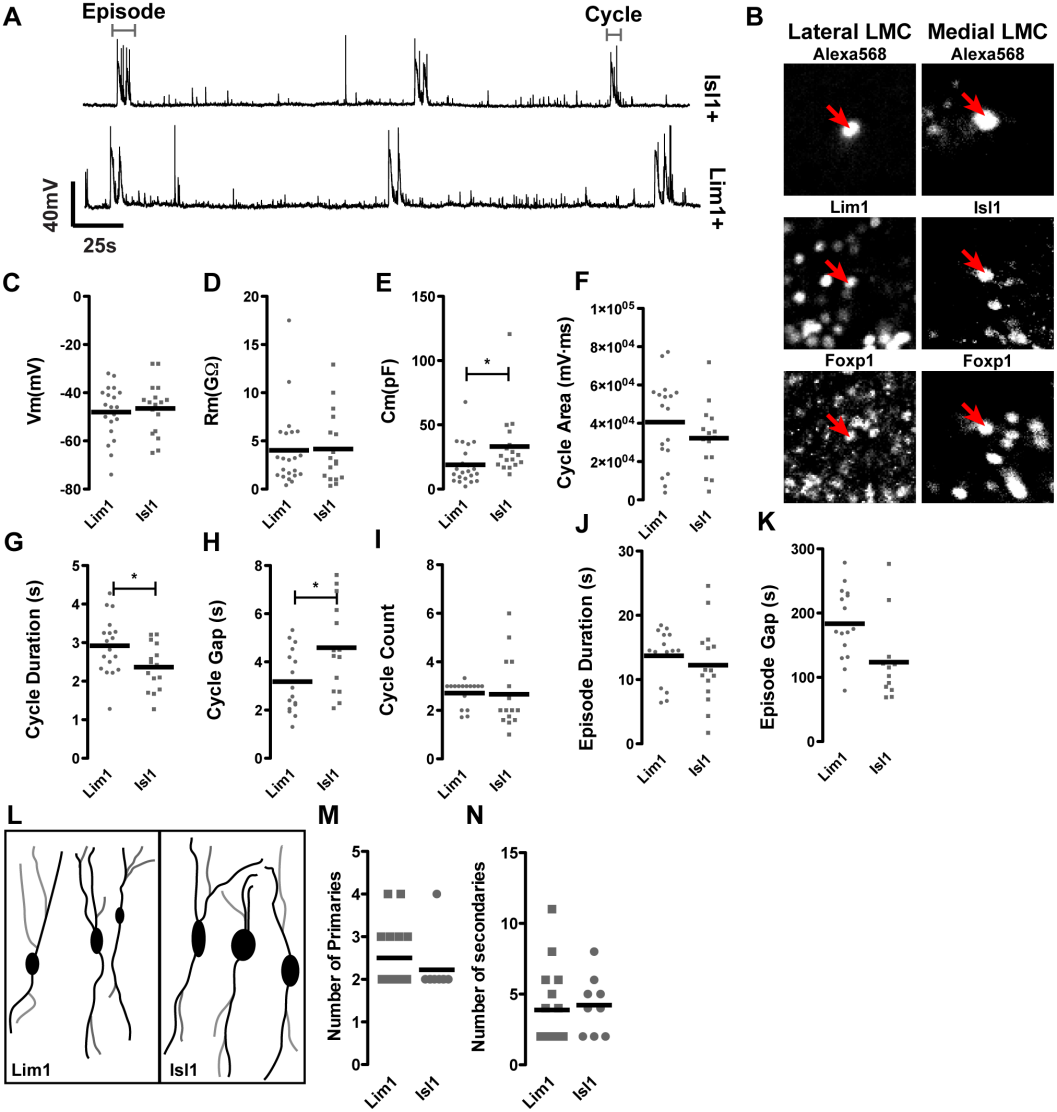
Electrical properties of Lim1 and Isl1-positive LMC neurons. A) Overall electrical activity profiles of Isl1^+^ and Lim1^+^ LMC subpopulations at HH St. 29/30 are similar; traces shown are recorded from two different preparations. B) Neurons filled with Alexa 568 dye are identified by post-recording immunostaining with Lhx1, Isl1 and Foxp1 antibodies. C) Comparison of membrane potential and membrane resistance (D) reveals both of these characteristics to be similar between cell types, whereas the membrane capacitance is higher in Isl1^+^ neurons (E). The magnitude of individual cycles is no different between cell types (F), though cycles are longer in Lim1^+^ cells (G) and there is a shorter interval between cycles in Lim1^+^ cells (H). The average number of cycles in an episode is not different (I), nor are episodes (J) or the intervals between episodes (K) longer in one cell type than another. Camera lucida tracing of filled neurons (L) allows counts of primary (black, M) and secondary (grey, N) processes, demonstrating no difference in branch characteristics of Isl1^+^ and Lim1^+^ neurons at the stages examined.

The neurotransmitter receptor genes *CHRNA5*, *CHRNA7, GRIK1, GRIN2A, HTR1A* and *HTR1B*, along with the KCC2 transporter are differentially expressed between medial (Isl1^+^) and lateral (Lim1^+^) LMC neurons from HH St. 24 onward. These differences in gene expression may contribute to the differential patterns of activity at later stages (E8; HH St. 33 onwards), such that medial and lateral motor neurons, innervating antagonistic muscles, fire in alternating episodes of activity due to the inhibitory effects of shunting chloride conductances [21], [29], [39]. We reasoned that such electrophysiological differences may already be apparent at earlier stages (HH St. 29/30). It has been previously demonstrated that differential expression of neurotransmitter receptor subunits alters the responses of functional channels to agonists [40], [41], affecting such characteristics as channel desensitization and sensitivity, which would functionally result in differences in the onset and duration of the rhythmic activity seen during normal motor circuit firing [4], [27]. To determine if medial and lateral LMC neurons had different firing properties, we dissected spinal cords from HH St. 29–30 embryos, then laid them lateral side up (“open-book”) in a chamber perfused with oxygenated Tyrode’s solution, then performed current-clamp recordings on single LMC motor neurons, which were identified post-hoc, through the expression of Foxp1, an LMC neuron identity marker. By combining the post-hoc analysis with detection of Isl1 or Lim1 transcription factors, this analysis allowed us to examine the temporal characteristics of electrical activity of individual medial and lateral LMC neurons (Fig. 2B).

Recordings from medial LMC and lateral LMC neurons in HH St. 29/30 spinal cords show similar patterns of activity, with regular depolarisations (‘cycles’) of 2.70±0.13s in length occurring every 152±12.82s interspersed with smaller depolarisations (<1s in length) at irregular intervals. (Fig. 2A). Eventually, the flexor and extensor muscle innervating motor neurons comprising these two populations exhibit different activity patterns. To uncover potential subtle electrophysiological differences between them, we measured their various electrical parameters. Analysis of resting membrane potential (Fig. 2C) and membrane resistance (Fig. 2D) shows no difference between medial and lateral LMC neurons (P>0.05 in both cases), indicating that their resting states are similar, though the capacitance of medial LMC neurons is higher than that of lateral LMC neurons (Fig. 2E; P<0.05), likely due to their greater size [42], [43]. Whilst several membrane properties are similar between medial and lateral LMC neurons, comparison of the voltage traces of these motor neurons during circuit activity reveals differences. The length of individual cycles in lateral LMC neurons is greater than in medial LMC neurons (Fig. 2G, P<0.05), and the gap between these cycles within an episode is shorter in the lateral population (Fig. 2H; P<0.05), though the number of cycles within an episode is not different (Fig. 2I; P>0.05), nor is the gap between episodes (Fig. 2H, P>0.05). These results are summarized in Table 2.

**Table 2:** Summary of characteristics of lateral and medial LMC neurons.

	Lim1	Isl1	P
**Vm (mV)**	−48.83±2.543	−47.02±2.543	>0.05
**Rm (GΩ)**	4.03±0.845	4.16±0.89	>0.05
**Cm (pF)**	18.91±3.327	28.78±2.973	**<0.05**
**Cycle Area (ms.mV)**	40591±5331	33768±4481	>0.05
**Cycle Duration (s)**	2.92±0.18	2.41±0.16	**<0.05**
**Cycle Gap (s)**	3.18±0.33	4.60±0.50	**<0.05**
**Cycle Count**	2.52±0.18	2.78±0.38	>0.05
**Episode Duration (s)**	12.37±1.277	13±1.49	>0.05
**Episode Gap (s)**	169.2±15.73	123.9±19.9	>0.05
**Primary Count**	2.50±0.18	2.22±0.22	>0.05
**Secondary Count**	3.88±0.68	4.22±0.68	>0.05

Given that medial LMC neurons are born prior to lateral LMC neurons [34], these subpopulations may have differentially matured patterns of connectivity leading to different activity patterns. We thus assayed the morphology and neurite branching of medial and lateral LMC neurons at HH St. 29/30 by imaging the dye-filled neurons immediately after recording, by which time neuronal processes could be seen emanating from the motor neuron cell bodies. However, counting the number of primary (processes that emanate directly from the soma) and secondary (processes that emanate from primary branches) processes revealed no significant differences between medial and lateral LMC neurons (Fig. 2L–N, Table 2; P>0.05).

These data provide the first demonstration of single-neuron activity in the early motor circuit, and demonstrate measurable differences between Isl1^+^ and Lim1^+^ LMC neurons that may arise as a consequence of differential NT receptor expression in these two sub-populations of neurons.

### Pharmacological Characterization of Individual Motor Neurons

It has previously been reported that the cholinergic and GABA-ergic neurotransmitter systems are most critical to total motor circuit activity at the earliest stages of its development [4], [27]. To relate the differential expression of neurotransmitter receptors in neurons of the medial and lateral LMC to the different firing characteristics of these neurons, we investigated the contribution of cholinergic, GABA-ergic and serotonergic neurotransmitter systems to individual motor neuron firing. Whole-cell patch-clamp recordings of LMC motor neurons at HH St. 30 allowed us to assay the membrane potential responses of single neurons to agonists and antagonists of cholinergic and GABA-ergic receptors with high temporal resolution. Pharmacological intervention in the motor circuit would allow us to examine the influences of the GABA-ergic, cholinergic and serotonergic neurotransmitter systems upon the firing of individual motor neurons on a cycle-by-cycle basis, revealing the characteristics of normal circuit firing that are mediated by these transmitter systems. To identify motor neurons, plasmids encoding EGFP driven by the Hb9 promoter (*Hb9-EGFP*;[44] were electroporated into the chick spinal cord at HH St. 18/19 and the electrical activity of labelled neurons in the LMC was assayed at HH St. 30 by single-neuron patch clamp in open-book preparations, as described above. Other than counts of cycles per episode, all data in this section are presented as a percentage, relative to baseline recordings and compared to this baseline.

**Figure 3:**
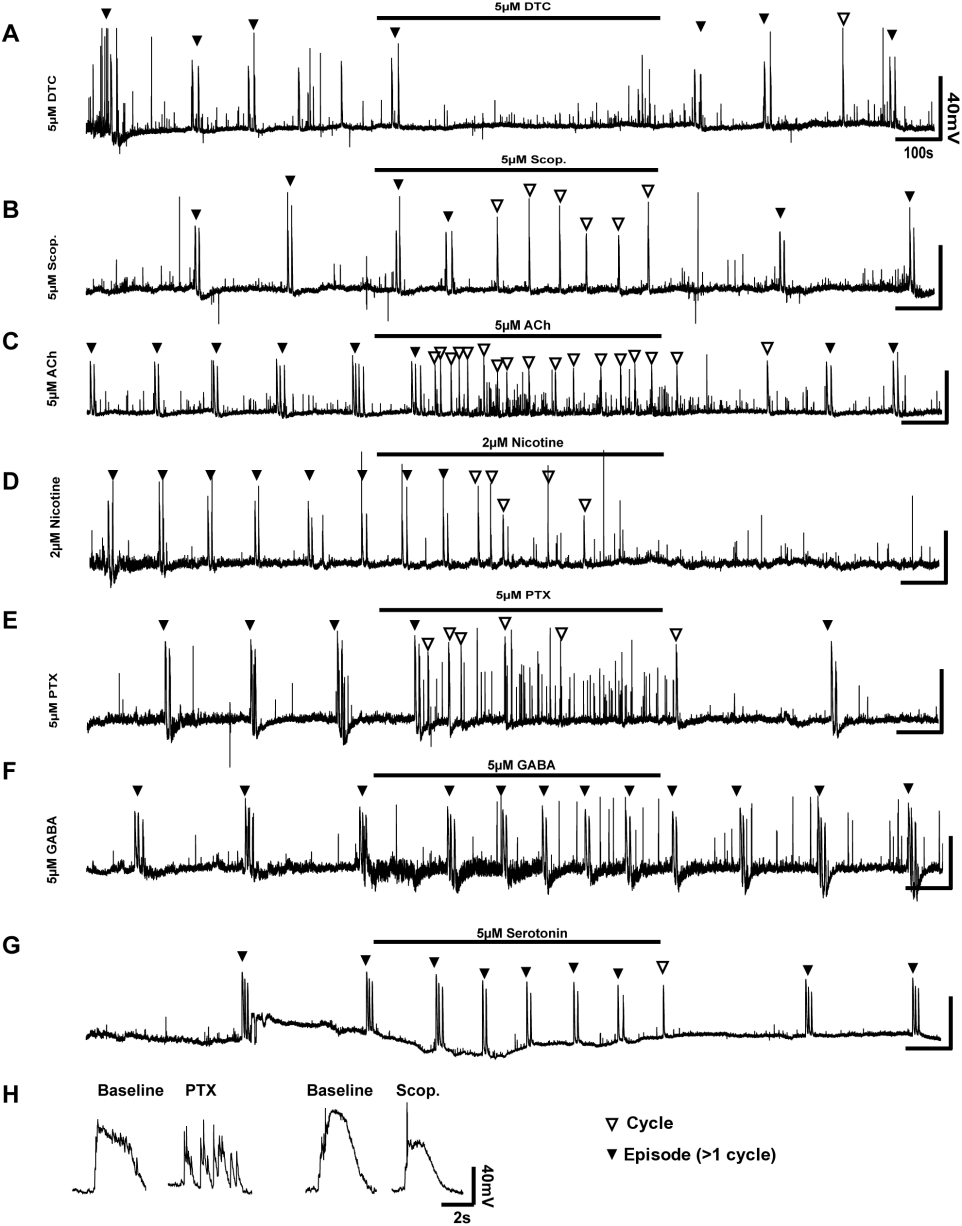
Pharmacological interrogation of individual motor neuron activity. 30-clamp recordings were made from HH St. 30 motor neurons identified by Hb9-EGFP, during which time agonists or antagonists were applied. A) Inhibition of nicotinic transmission with 5 μM D-Tubocurarine (DTC) completely abolished episodic activity (black arrowheads). The activity seen at the end of the treatment does not fulfil the criteria for a cycle. B) Muscarinic inhibition with 5 μM scopolamine (Scop.) resulted in changes in episode patterns, with an abolition of doublet episodes (black arrowheads) and an increased frequency of singlet cycles (white arrowheads). C) Nicotinic stimulation (2 μM) completely abolished the pattern of activity; irregular singlets were seen throughout the drug application, and many more small depolarisations also occurred. Activity did not return after cessation of nicotine application. D) Stimulation with acetylcholine (5 μM) increased the frequency of both singlet cycles, and short depolarisations. E) GABA-A inhibition with Picrotoxin (PTX, 5 μM) abolished episodic activity; irregular singlet cycles remained, interspersed with many short depolarisations that do not fit the criteria of cycles. F) GABA application (5 μM) increased the frequency of episodic activity without causing significant changes to episode structure. G) Application of 5 μM Serotonin increased the frequency of episodic activity, and reduced the number of cycles in episodes. H) The shape of voltage traces for a typical individual cycle in baseline conditions compared to a typical cycle in the presence of PTX demonstrate the disruption to cycles in the absence of GABA-A signaling.

At HH St. 30, all neurons from which we recorded showed robust activity, similar to that described in the previous section, with ‘episodes’ of cycles occurring every few minutes; small depolarisations could be detected during these intervals between episodes. Application of the nicotinic receptor antagonist D-Tubocurarine (DTC) had clear effects upon motor circuit firing. While perfusion with 2 μM DTC had no significant effects upon either the frequency of motor neuron firing (inter-episode interval = 118.1±43.1%; P>0.05) or cycle length (cycle length = 94.5±15.5%), DTC application at 5 μM completely blocked cycles, leaving only small depolarisations (Fig. 3A). These results demonstrate that nicotinic transmission is absolutely required for the firing of the motor circuit, as might be expected given the widespread distribution of CHRNA channels in the spinal cord. In contrast to DTC, application of the muscarinic antagonist scopolamine (5 μM, Fig. 3B) increased the frequency of motor neuron firing (inter-episode interval = 49.7±26.1%; P<.0001; Fig. 3B) and reduced the length of cycles (cycle length = 91.9±14%; P<0.05). Application of the cholinergic agonist acetylcholine (ACh, 5 μM, Fig. 3C) lead to excitation of the firing pattern during the period of drug application; organized episodes were largely ablated in the presence of ACh, though cycles were present at higher frequency than in baseline recordings (inter-cycle interval = 30.7±12.9%). Nicotine stimulation (2–5 μM, Fig. 3D) also caused an apparent increase in the frequency of cycles (inter-cycle interval = 90.04±24.2%, P<0.05), though to a lesser degree than ACh (P<0.0001). Episodic activity did not re-appear after 10 minutes of washout.

The GABA-ergic system is also known to be critical for the early activity of the motor circuit [4], [27]. Application of the GABA-A antagonist picrotoxin (PTX, Fig. 3E) at 5–10 μM resulted in a disruption of episode pattern: early in the application, neurons fired normal, sustained cycles; over time, these cycles became separated from sustained cycles into multiple short depolarisations that lasted for a length of time similar to a baseline cycle (cycle length = 88.6±26.02%, P>0.05; Fig. 3E and H). Additionally, the frequency of cycles was reduced by 50.72±15.3% (P<0.01). Application of GABA (2–5 μM, Fig. 3F) lead to an increase in the high-frequency, low amplitude oscillation (‘noise’) of the resting membrane potential by 175.1±17.8% (P<0.05), and an increase in the frequency of episode firing (inter episode interval = 75.8±32.6%, P<0.05), though the number of cycles per episode remained similar (2.75±0.77 cycles/episode in baseline vs. 2.75±0.75 cycles/episode in GABA-treated embryos, P>0.05).

Serotonin stimulation is known to induce motor circuit firing in neonatal rat spinal cords [45], [46]. Whilst we could not detect *SERT-1* mRNA in the spinal cord at HH St. 30, we were able to detect mRNAs encoding serotonin receptors (Fig. 1E–F, Table 1; data not shown), leading us to examine if Serotonin application at early stages could also induce circuit firing. At HH St. 30, application of Serotonin (5–10 μM, Fig. 3G) lead to an increase in the frequency of episode firing (episode interval = 57.3±25.4%, P<0.001; Fig. 2H), and a reduction in the number of cycles per episode (from 2.375±0.72 cycles/episode to 1.35±0.56, P<0.001) demonstrating that serotonin plays an excitatory role in the early motor circuit.

Together, these data demonstrate that the cholinergic, GABA-ergic and serotonergic systems are excitatory at HH St. 30, though the inhibition of muscarinic receptors with scopolamine was also excitatory, suggesting a more complex role for muscarinic stimulation in motor circuit firing.

### Pharmacological Characterization of Motor Neuron Populations

The above data demonstrate the effects of various neurotransmitter systems upon single motor neurons with very high temporal resolution, but do not provide any information about the response of populations of motor neurons to pharmacological manipulation. We thus supplemented our electrophysiological recordings with data from calcium imaging, allowing analysis of large populations of motor neurons simultaneously (mean 24.2±1.75 neurons/field of view, maximum 64). Plasmids encoding EGFP driven by the Hb9 promoter (*Hb9-EGFP*) were co-electroporated into the chick spinal cord at HH St. 18/19 with plasmids encoding the R-GECO calcium indicator [47], and the activity of LMC neurons was imaged at HH St. 30 using a spinning disk confocal microscope. Baseline recordings were made for 10–20 minutes prior to the application of drugs; imaging was performed during drug treatment for 20 minutes, before washout. All data were analysed using the automated cycle detection routine described below, and normalized to baseline recordings made in that embryo.

**Figure 4:**
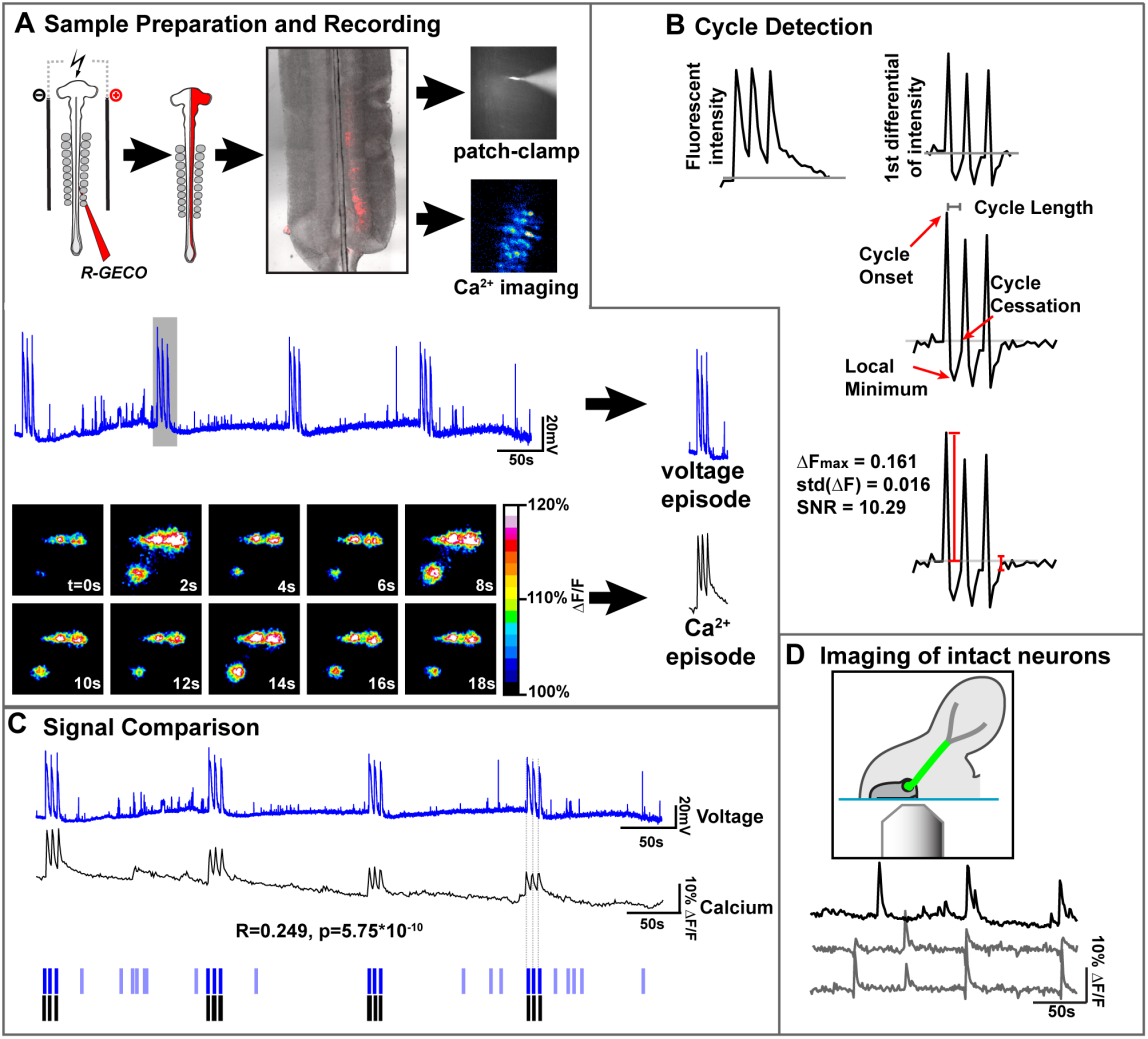
Calcium imaging reflects the electrical activity of spinal motor neurons. A) Co-electroporation of *RGECO* and *Hb9-GFP* expression plasmids labels LMC motor neurons which can be seen in an open book preparation at HH St. 30. Single motor neurons were simultaneously patch-clamped and imaged, showing calcium transients that correspond to voltage cycles. Peaks in voltage recordings are mirrored in calcium traces. B) The cycle detection routine transforms raw calcium traces into their first differential (1°∂). When the 1°∂ is >2 s.d from the mean, an cycle is detected that lasts until the first time the 1°Δ reaches 0 after passing a local minimum. Signal to noise ratio (SNR) is also calculated. C) Comparison of voltage and calcium recordings shows a very significant correlation between the two. Critically, our cycle detection routine (B) detects the onset of calcium cycles that accurately reflect cycles in the voltage trace. D) Imaging neurons with intact axons shows that activity patterns are similar in intact preparations (grey lines) compared to open-book preparations (black lines).

We first ascertained whether the LMC neuron changes in calcium concentration detected by our calcium imaging regime reflect accurately the changes in membrane potential detected by whole-cell patch-clamp recordings (Fig. 4). Spinal cords electroporated with *R-GECO* were dissected at HH St. 30 into an open-book configuration (Fig. 4A), then large, bipolar neurons located within the LMC region expressing R-GECO were patch-clamped, and simultaneous recordings of membrane potential and R-GECO intensity were made for 10 minutes, and the signals from these two methods compared (Fig. 4A). To do this, we developed an automated cycle detection routine using MATLAB. Briefly, this routine looks for large changes in calcium signals (Fig. 4B) and marks these as cycle onsets (Fig. 4B, 4C). The duration of such cycles is taken as being the time difference between the onset and the first time when the differential returns to 0 from negative values (Fig. 4B). Using results from this algorithm, we compared voltage and calcium signals over 10 minutes (Fig. 4C, C) for 4 neurons from 4 embryos. 47/48 cycles detected in calcium traces were reflected in the voltage traces (mean accuracy = 97.91±4.17%), demonstrating that our methods for detecting calcium activity accurately reflected voltage activity. Similar results were obtained from 11 neurons labelled with fluo-4-AM (fluo4; accuracy = 95.47±11.9%). However, it must be noted here that many shorter events (mean length = 421.6±137.6 ms) detected in voltage traces were not detected using calcium imaging (Fig. 4C, pale blue bars), indicating that these events either do not enable calcium entry into firing neurons, that the corresponding increase in calcium indicator intensity is too small to be detected using the method described here, or that the visual sampling rate is too low to detect such events. Comparison of cycle length between electrophysiology and calcium imaging reveals that cycles recorded from *R-GECO* electroporated spinal cords are 92.1±54.2% longer relative to the same cycle recorded by electrophysiology (P<0.0005). This discrepancy is likely due to either calcium dynamics, or the properties of the R-GECO sensor. The time from cycle onset to peak fluorescence in R-GECO-visualized cycles was ∼1s, the limit of the temporal resolution used in these experiments. The time from the last peak in an episode to the return to baseline represented 53.6±17.8% of the total length of the recorded episode. Such differences in cycle length preclude the quantitative comparison of cycle lengths between data recorded by electrophysiological and calcium imaging means, though it is clear that cycle onset is accurately recorded by calcium imaging.

**Figure 5:**
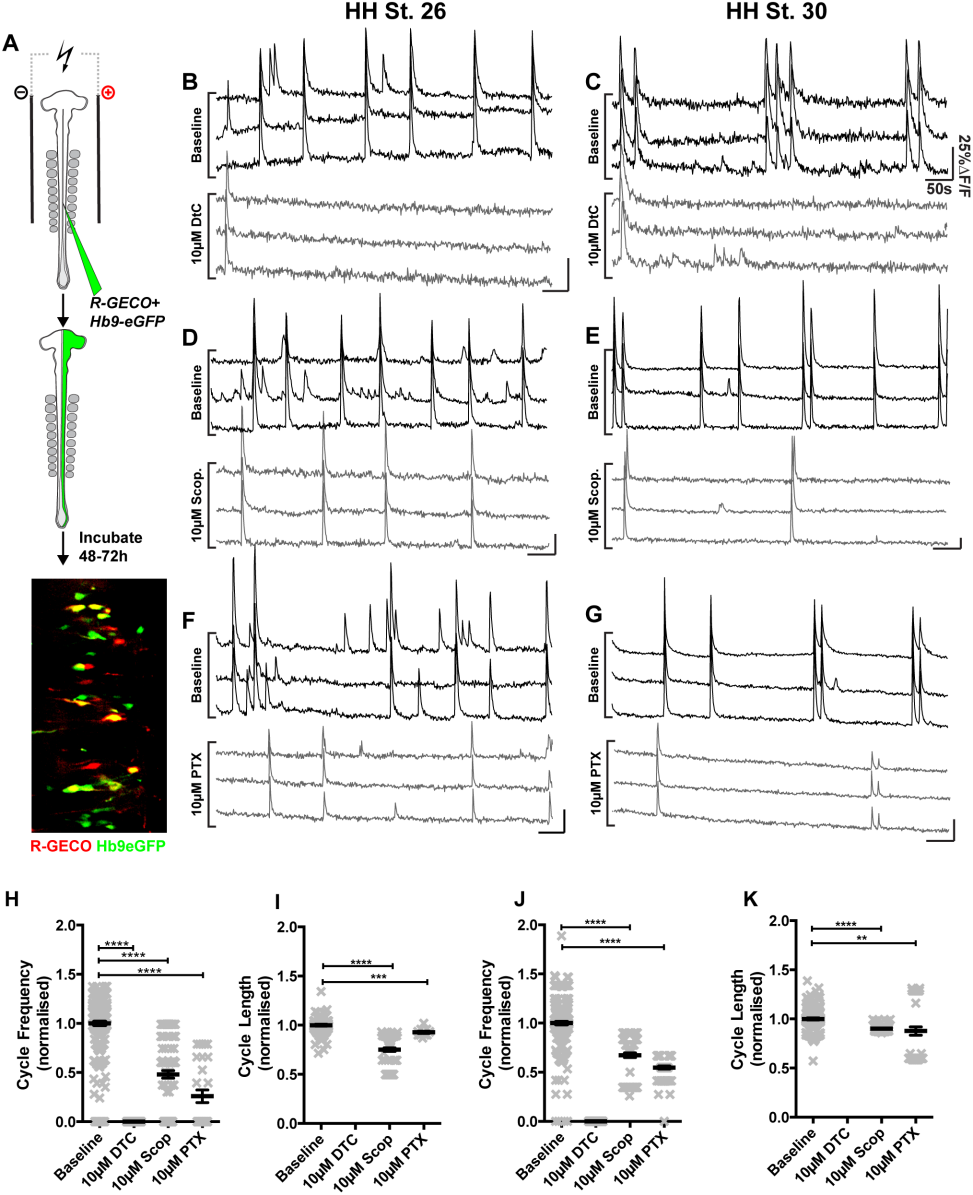
Pharmacological interrogation of motor neuron population activities. A) Co-electroporation of *Hb9-GFP* and *R-GECO* expression plasmids, followed by dissection and calcium imaging reveals patterns of activity in LMC motor neurons. B) Application of the nicotinic antagonist D-Tubocurarine (DTC, 10 μM) to HH St. 26 spinal cords blocks detectable activity. C) 10 μM DTC application blocks activity at HH St. 30. D–E) Application of the muscarinic antagonist Scopolamine (Scop., 10 μM) at HH St. 26 (D) and HH St. 30 (E) reduces the frequency of firing. F–G) Application of the GABA-A antagonist picrotoxin (PTX, 10 μM) at HH St. 26 (F) and HH St. 30 (G) reduces the frequency of firing. H) Cycle frequency at HH St. 26 in the presence or absence of blockers. I) Cycle length at HH St. 26 in the presence or absence of drugs. J) Cycle frequency at HH St. 30 in the presence or absence of blockers. K) Cycle length at HH St. 30 in the presence or absence of drugs. These comparisons are summarized in Tables 2 and 3.

One possible origin of the neuronal activity in our preparation was damage to neurons caused during their dissection. To examine the activity patterns of neurons with intact axons, we split *R-GECO* electroporated embryos down the midline, leaving motor axons intact, and imaged motor neuron activity through the lumenal face of the spinal cord (Fig. 4D). The activity profile in these preparations was similar to that in our *ex vivo* preparations (Fig. 4D), suggesting that calcium imaging of motor neurons in an open-book preparation reflects the electrical activity of healthy motor neurons. Thus, co-electroporation of plasmids encoding R-GECO with Hb9-eGFP (Fig. 5A), along with selective imaging of the LMC allows specific detection of calcium changes in motor neurons.

Application of 10 μM DTC at both HH St. 26 and HH St. 30 (Fig. 5B and C) resulted in a complete blockade of detectable activity (Fig. 5B, C, H–K), though application of 5 μM DTC had no effect upon either frequency or length of cycles in motor neurons at HH St. 30 (Tables 3 & 4, P>0.05 for each comparison). The likely cause for discrepancy between these results and those obtained in the corresponding electrophysiological experiments (Fig. 3A) are differences between the recording chambers used for each experiment (see methods), and the nature of the effect of DTC upon motor neuron firing, where sub-threshold concentrations produce no effects. Application of DTC at higher concentrations (10 μM) completely abrogates calcium activity in Hb9^+^ motor neurons and Hb9^−^ interneurons (100% reduction in frequency; Tables 3 & 4 and data not shown). Application of 2 μM scopolamine at HH St. 30 significantly increased the frequency of cycles (185.8%; Table 2), and reduced the length of cycles by (31.2%; Table 3). The increase in frequency induced by low doses of scopolamine is largely comparable to electrophysiological recordings where it rose to 201±26%. Conversely, application of 10 μM scopolamine reduced the frequency of cycles by 51.9% at HH St. 26 and 32.7% at HH St. 30 (Fig. 5 H & J, Table 3); the length of cycles was reduced by 25% at HH St. 26 and by 10% at HH St. 30 (Fig. 5D, E, I & K, Table 4).

**Table 3:** Analysis of normalized cycle frequency during drug application.

Group A				Group B				
Condition	n	Mean	S.E.M	Condition	n	Mean	S.E.M	P
HH St. 26 Baseline	75	1.000	0.037	HH St. 26 10 μM DTC	50	0.000	0.000	<0.0001
HH St. 26 Baseline	98	1.000	0.021	HH St.26 10 μM Scopolamine	94	0.481	0.039	<0.0001
HH St. 26 Baseline	29	1.000	0.056	HH St. 26 10 μM PTX	28	0.259	0.064	<0.0001
HH St. 30 Baseline	43	1.000	0.081	HH St. 30 10 μM DTC	45	0.000	0.000	<0.0001
HH St. 30 Baseline	90	1.000	0.013	HH St. 30 2 μM Scopolamine	88	1.858	0.039	<0.0001
HH St. 30 Baseline	86	1.000	0.006	HH St. 30 10 μM Scopolamine	81	0.6733	0.024	<0.0001
HH St. 30 Baseline	69	1.000	0.010	HH St. 30 10 μM PTX	65	0.5477	0.016	<0.0001

n = number of neurons examined; ≥3 embryos examined per condition.

**Table 4:** Analysis of normalized cycle length during drug application.

Group A				Group B				
Condition	n	Mean	S.E.M	Condition	n	Mean	S.E.M	P
HH St. 26 Baseline	110	1.000	0.005	HH St.26 10 μM Scopolamine	92	0.750	0.019	<0.0001
HH St. 26 Baseline	28	1.000	0.013	HH St. 26 10 μM PTX	11	0.928	0.013	0.003
HH St. 30 Baseline	90	1.000	0.01	HH St. 30 2 μM Scopolamine	88	0.689	0.021	<0.0001
HH St. 30 Baseline	86	1.000	0.004	HH St. 30 10 μM Scopolamine	81	0.901	0.004	<0.0001
HH St. 30 Baseline	69	1.000	0.003	HH St. 30 10 μM PTX	64	0.877	0.043	0.005

n = number of neurons examined; ≥3 embryos examined per condition.

Inhibition of GABA-A signaling by application of 10 μM PTX resulted in a reduction of cycle frequency at both HH St. 26 (74.1%, Fig. 5F & H, Table 3) and HH St. 30 (45.2%, Fig. 5G & J, Table 3), as well as a small reduction in cycle length (7% at HH St. 26; 12% at HH St. 30; Fig. 5I, K; Table 4). PTX-induced changes in frequency derived from calcium imaging are comparable to those derived from electrophysiological recordings at HH St. 30 (50.72±15.3%).

Here, we demonstrate a robust method for simultaneous imaging of calcium activity in large populations of individual neurons. Whilst significant quantitative differences exist between the calcium imaging data and electrophysiological recording data, our experiments argue that optical recordings of Calcium fluxes are an accurate proxy for current recordings. These data show that from very early stages of motor circuit activity, neurons fire under the influence of muscarinic, nicotinic and GABA-ergic circuitry, with nicotinic transmission being critical to the maintenance of motor neuron activity at early stages of development, while muscarinic and GABA-ergic signaling play more modulatory roles. Recording activity patterns from HH St. 26 and HH St. 30 embryos reveals differences between these stages, suggesting that motor neuron activity patterns re-organize through time.

### Development of Activity in Motor Neurons

**Figure 6:**
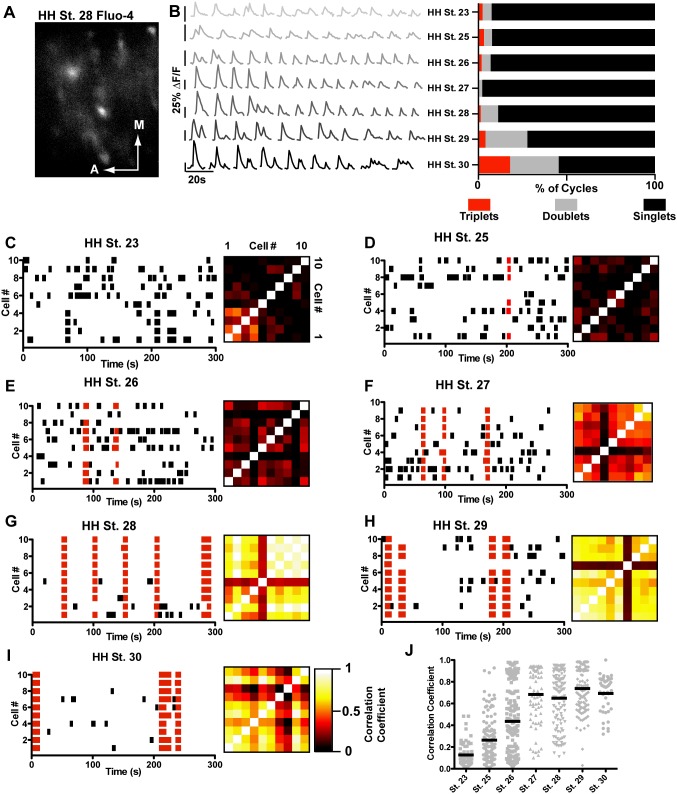
Synchronized activity develops over time. A) Example of a field of view from an HH St. 28 embryo containing fluo-4 labelled motor neurons (red dotted regions, scale bar = 100 μM). B) Examples of calcium traces from single cells through development; cycles become less variable over time, and regularly fire in episodes of multiple cycles. C–I) Raster plots and correlograms of activity in populations of cells between HH St. 23 and HH St. 30. Black bars indicate uncorrelated cycles, in which <60% of the motor neurons in the field of view participate, red bars indicate correlated cycles, in which >60% of motor neurons participate. The firing pattern of neurons is initially apparently random, becoming more synchronous with time such that by HH St. 28, very few cycles occur in an asynchronous fashion. Similarity of activity patterns between two given neurons is represented by black-framed correlograms that increase in brightness over time, representing the increase correlation between cells. J) Mean correlation coefficient of neurons over time increases from HH St. 23 to HH St. 30, as the firing of individual neurons in the circuit becomes more synchronous.

We next examined activity patterns in motor neurons at a very early stages of motor circuit assembly and how these patterns develop over time. Retrograde labelling of motor neurons using injections of the calcium indicator fluo-4-AM injected into the limb, allows robust, acute and specific labelling of motor neurons (Fig. 6A). This method allowed imaging of calcium transients in many motor neurons simultaneously, providing information about activity in both individual motor neurons, and populations of such. Given that motor neurons are the only lumbar spinal neurons projecting axons into the periphery at the stages examined (dorsal root ganglia are removed), and that fluo-4-AM is hydrolyzed in the cytoplasm to fluo-4 [48] rendering it unable to re-cross the cell membrane, we were confident that our approach was labelling motor neurons. Activity was recorded using fluo-4, the accuracy of which was verified by electrophysiology at HH St. 30, similar to the experiments described for *R-GECO* above (Fig. 4; fluo-4 onset accuracy = 95.47±11.9%, 11 neurons). Cycles recorded optically were 207.9±34.7% longer than the same cycle recorded electrophysiologically. The onset of cycles recorded by fluo-4 was similar to *R-GECO* (∼1 s from baseline to peak), but the return to baseline from peak fluorescence was slower (66.1±6.8% in fluo-4 vs 53.6±17.85% in *R-GECO*; P<0.05). These experiments allowed us to detect large populations of motor neurons from very early stages of motor circuit formation, and analyze their activity patterns to characterize the changes in both individual and population activity over time.

**Figure 7:**
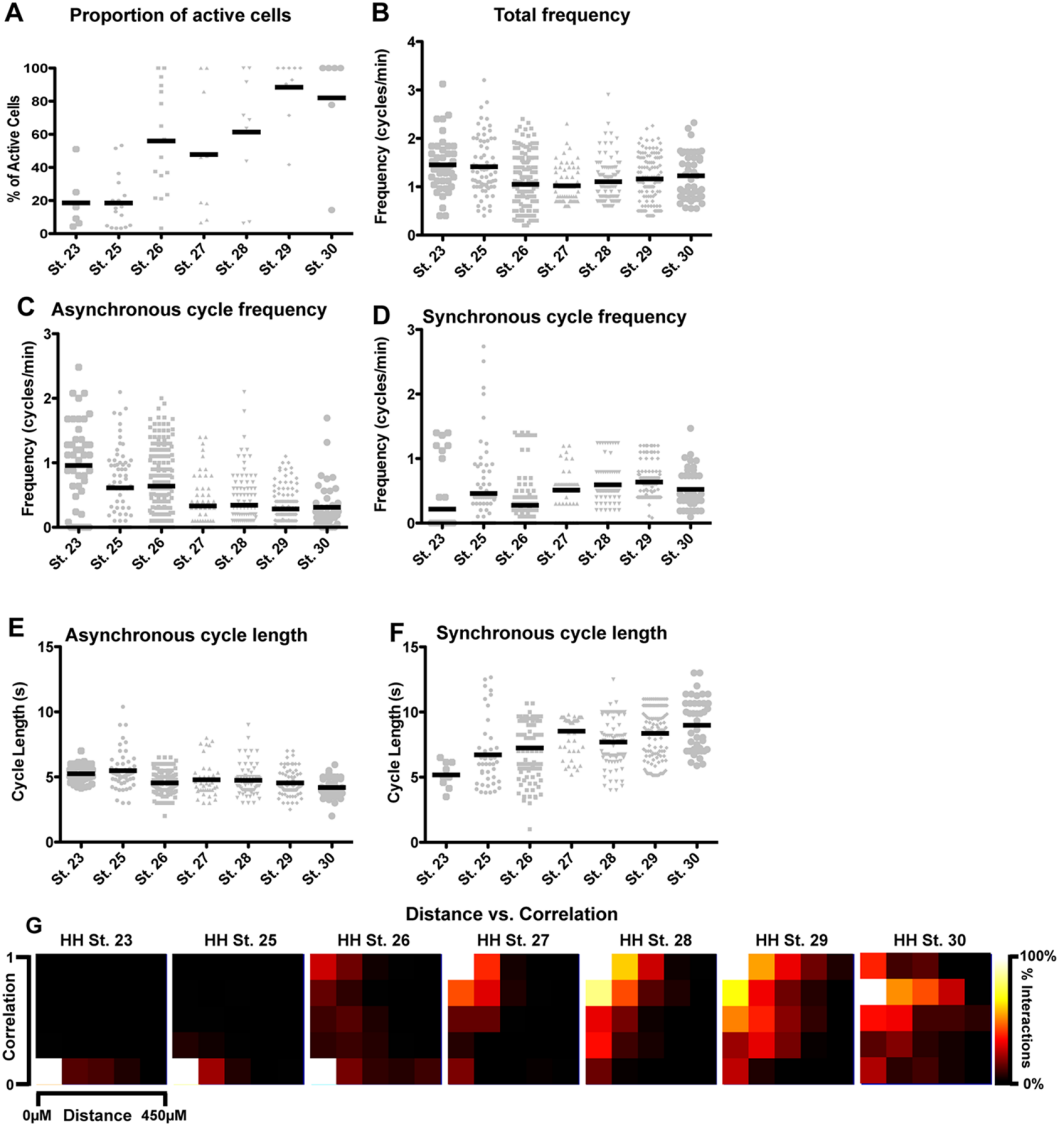
Variation in the synchronicity of activity with developmental age and neuron-neuron distance. A) Over time, the percentage of active neurons in a preparation increases from 18.61%±7.19% to 81.01±14.02%; p<0.0001, one-way ANOVA. B) The frequency of motor neuron firing shows significant changes over time, decreasing moderately from HH St. 23 to HH St. 30. C) There is a decrease in the frequency of asynchronous cycles over time counteracted by only a small increase in the frequency of synchronous cycles (D). E) The length of asynchronous cycles decreases, whilst the length of synchronous cycles increases over time (F). G) Analysis of the distance between two given cells and the correlation between them shows that embryos at HH St. 23 and HH St. 25 are dominated by asynchronous cycles, but that older embryos have higher synchronicity over longer distances. Pixel intensity indicates the percentage of interactions at a given correlation and distance.

Analysis of individual motor neurons at each stage reveals that the characteristics of calcium transients changes over time. At HH St. 23–25, cycles occur seemingly randomly within neurons; the shape of cycles is very variable within individual neurons, and other neurons within the same preparation. Episodes (defined as doublet or triplet cycles that occur after the primary cycle, prior to a return of fluorescent intensity to baseline) comprise 7.55% of all cycles at HH St. 23, and 7.78% of cycles at HH St. 25 (Fig. 6B, Table 5), though these occur only in single neurons (i.e. asynchronously). Between HH St. 26 and HH St. 28, transients become more regular in shape, though episodes (two superimposed cycles) are still rare (Fig. 6B, Table 5). At HH St. 29 and 30 transients are almost uniform in shape between cells; additionally doublets and triplets of activity are frequent (Fig. 6B, Table 5). These data are suggestive of a greater uniformity of activity at later stages of development, with older neurons being able to fire in episodes of activity with multiple cycles, while younger neurons fire cycles that are much more varied in shape and size. By pooling results from all recorded neurons at each timepoint, we can see that over time, a greater proportion of labelled neurons in each field of view is active (Fig. 7A; from 18.61±7.189% at HH St. 23 to 82.01±14.02% at HH St. 30, p<0.0001, one-way ANOVA). Additionally, there is a modest but significant decrease in total frequency over time (Fig. 7B, HH St. 23 frequency = 1.45±0.082 cycles/min, N = 44; HH St. 30 frequency = 1.228±0.073 cycles/min P<0.05).

**Table 5:** Cycles occurring in active neurons through development.

Developmental Stage	N (embryos)	n (active neurons)	ne (cycless)
HH St. 23	3	44	741
HH St. 25	4	48	809
HH St. 26	9	185	1968
HH St. 27	6	60	597
HH St. 28	6	102	1023
HH St. 29	6	117	1757
HH St. 30	3	44	764

It has previously been demonstrated that motor neurons synapse upon each other [27], [49], [50], increasing the probability of connected motor neurons firing together. We reasoned that if this were the case, motor neurons that had been connected together for longer periods would be more synchronous, and as they connected to more motor neurons, this synchronicity would spread among connected neurons. Given that we see motor neuron populations firing synchronously (i.e. firing multiple cycles at the same time as other neurons) at HH St. 30 (Fig. 5), we hypothesized that at the earlier stages examined, we would be able to see more asynchronous neuronal activity. We thus performed various measures of synchrony on our calcium imaging data. Initially, we modified our cycle detection routine to detect cycles that occur simultaneously in >60% of neurons in a field of view, and divided cycles into either synchronous (Fig, 6C–I, red bars) or asynchronous (Fig, 6C–I, black bars). With this analysis, we can see that through development, calcium cycles go from highly asynchronous and apparently random at HH St. 23 (Fig. 6C) to almost completely synchronous by HH St. 28–30 (Fig. 6G–I). To examine the synchronicity in a quantitative fashion, we calculated the correlation coefficient of firing between neurons in each field of view at each stage, allowing us to examine how closely each pair of neurons in a field of view fired together; if synchronicity is increasing over time, we would expect to see pairs of neurons with higher correlation coefficients at later stages than at earlier stages. This analysis reveals a general increase in synchronicity between HH St. 23, where the entire correlogram (black framed boxes; each pixel representing the interaction between two cells: values of 1, white, represent complete synchrony; values of 0, black, represent no synchrony) is very dark, to a very bright correlogram at HH St.30. Calculation of each neuron’s mean correlation coefficient shows that over time, neurons go from being asynchronous (Fig. 1J, HH St. 23, mean correlation coefficient = 0.1267±0.011) to more highly synchronous (HH St. 30, mean correlation coefficient = 0.69±0.024; p<0.0001).

An increase in synchronicity over time could result from several factors: a decrease in the frequency and/or length of asynchronous cycles; or an increase in the frequency and/or length of synchronous cycles. To examine which of these factors contributed to the increase in synchronicity seen in Fig. 6J, we split cycles into synchronous or asynchronous as above, then performed analysis of length and frequency on these independent groups. This analysis reveals a significant downward trend in the frequency of asynchronous cycles (p<0.0001, one-way ANOVA) and a significant upward trend in the frequency of synchronous cycles (p<0.0001, one-way ANOVA), with 79.5% of active cells at HH St. 23 never firing in a synchronous fashion (data not shown). The length of synchronous cycles increases over time, (p<0.0001, one-way ANOVA) consistent with an increase in overall correlation, whereas the length of asynchronous cycles slightly decreases over time (p<0.0001, one-way ANOVA). These results suggest that the increase in synchronicity through development is due to the conversion of short cycles in single neurons into longer cycles in which many neurons participate.

This conversion from short single-neuron cycles to longer multi-neuron cycles may arise as a result of individual neurons recruiting their neighbours to fire alongside them; given that all the neurotransmitter systems we examined appeared to be functionally excitatory at HH St. 30, and the existence of reciprocal motor neuron connections [27], [49], it seems highly plausible that this is the case. To examine this, we performed a comparison of the distance between two neurons in a field of view and the level of correlation between them. If neurons were recruiting their neighbours to fire with them, we would expect to see that correlation coefficients initially increase at short distances, then these correlated interactions spread over larger distances. Two-dimensional histograms plot distance on the X-axis and correlation coefficient on the Y-axis, with the bin size reflected by pixel intensity (Fig. 7G). It can be seen that at HH St. 23–25 the majority of interactions between two cells are very asynchronous (Fig. 7G) and occur over short distances. Between HH St. 26 and 27, a shift towards more synchronous cycles occurs, with the majority of synchronous cycles being at shorter distances. From HH St. 28 onward, highly synchronous interactions occur at longer distances than previously. These data demonstrate that neurons that are closer to one another begin to fire together earlier than neurons that are farther apart.

In summary, we can see that over time, greater numbers of neurons become active. The length and frequency of synchronous cycles increases over time, while the frequency and length of asynchronous cycles decreases over time, reflected in the changes in correlation coefficient. Examining the relationship between neuron-neuron distance and correlation shows that over time, neurons become more synchronous with partners over longer distances.

## Discussion

In this study, we have documented the expression of a wide array of neurotransmitters and neurotransmitter receptors found to be expressed in neurons within the early chick spinal cord and, at the level of individual motor neurons and populations thereof, assayed the influence of the major neurotransmitter systems that drive early activity in the chick motor circuit. We also use the first examples of patch-clamp recordings from early chick motor neurons to demonstrate that, despite the diversity of neurotransmitter receptors differentially expressed by medial and lateral LMC neurons, there are surprisingly few detectable differences in their firing patterns at the level of individual motor neurons during early stages of development. Finally, we describe the emergence of synchronous activity from the earliest stages of motor network activity. Here we discuss these findings in the context of neuronal diversity and the emergence of mature neuronal activity patterns.

### Differential Expression of Neurotransmitter Receptors in the Context of Early Circuit Activity

To ascertain the nature and temporal origin of the neuronal diversity that underlies differential activity patterns in the maturing motor circuit [29], we assayed the expression of neurotransmitter systems in the ventral spinal cord and compared activity recorded from motor neurons in the medial and lateral LMC. From the earliest stages of motor circuit activity, all five neurotransmitter systems examined in this study are competent to influence activity in the spinal cord, with GABA and acetylcholine driving circuit activity at the earliest stages [4], [27] this study). Several genes encoding cholinergic and GABA-ergic receptor subunits are differentially expressed between medial and lateral LMC neurons, including *CHRNA5*, *CHRNA7*, *and GABRA3*, which may contribute to the subtle differences in firing characteristics observed in these neurons. The specific expression of *CHRNA5* in medial LMC neurons is particularly intriguing, as this is a purely modulatory subunit, with no ACh binding capability [51]. The presence of CHRNA5 subunits in an alpha α3β2 receptor increases the rate of desensitization of the receptor, resulting in a shorter response to ACh [41], in line with our data demonstrating preferential expression of *CHRNA5* in Isl1^+^ medial LMC neurons (Fig. 1A) and their shorter cycle lengths (Fig. 2G). CHRNA5 subunits are also associated with increased sensitivity to ACh and increased permeability to Ca^2+^ [40], characteristics that may also lead to differences in firing between medial and lateral LMC neurons both individually and as populations. Lozada and Berg have previously seen that specific loss of CHRNA7 leads to reduced formation of glutamatergic synapses in hippocampal neurons [52], suggesting that selective expression of this gene in lateral LMC neurons may lead to increased formation of glutamatergic synapses in these neurons relative to medial LMC neurons. Additionally, CHRNA7 is critical for mediating the effects of nicotinic transmission upon the polarity of GABA signaling; in the absence of CHRNA7 signaling, GABA continues to exert excitatory effects for longer periods due to retention of NKCC1 expression and failure to increase levels of KCC2 protein [53], fitting with our observation that KCC2 levels appear lower on lateral LMC neurons, which express *CHRNA7*. Additionally, the relatively higher expression of KCC2 on medial LMC neurons compared to lateral LMC neurons (Fig. 1 G) suggests that medial LMC neurons are likely to have a higher [Cl^−^]_i_, and would thus be less excited by GABA, resulting in the shorter cycle durations and longer gaps between cycles seen in these neurons (Fig. 2G–H). The differential expression of *GABRA3* between medial and lateral LMC neurons will lead to different binding affinities of functional GABA receptors [54], resulting in different responses of these neurons to early neurotransmission mediated by GABA. Whilst there is as yet no evidence for glutamate and glycine acting to drive circuit activity in the early motor circuit, the repetitive firing of glutamatergic/glycinergic interneurons with motor neurons that express relevant neurotransmitter receptor mRNAs (i.e. *GRIK1*, *GRIN2A*, *GRIA1*, *GLRB*) is likely to induce selective patterns of connectivity that are critical for later patterns of motor activity, given the evidence for these neurotransmitter systems in synaptic remodeling in other systems [14], [55]–[59]. The differential expression of *HTR1A* and *1B* mRNAs between LMC subpopulations may also lead to differential modeling of dendritic trees; functionally, these G-protein-coupled receptors have been shown to have different levels of association to both adenylate cyclase and ERK2 responses [60], suggesting that these receptors may also regulate the morphology of dendritic trees, which are diverse between motor pools [59], [61], [62]. It is intriguing to note that Wolfram et al., have demonstrated, in Drosophila Melanogaster, that Isl1 negatively regulates the expression of the Shaker potassium channel, suggesting that this transcription factor could underlie the differential expression of a multitude of genes that lead to differential activity of medial and lateral LMC neurons in chick [63]. Studies in *X. laevis* from the Spitzer lab have demonstrated that in this species, homeostatic mechanisms exist to regulate neurotransmitter identity of spinal neurons [7], [64], such that reducing total calcium activity results in increased numbers of excitatory neurons. Whilst this mechanism does not appear to influence the transcriptional identity of spinal motor neurons in chick [12], the differences in activity patterns observed in medial and lateral LMC neurons in this study could in principle contribute to the differential expression of neurotransmitter receptors.

It has previously been reported that differences can be seen between the nerve root activity recorded from different motor pools in embryos as early as HH St. 25.5 [4], where it is observed that episodes of flexor activity are longer than extensor episodes, reflected by our observation that lateral LMC neurons fire longer cycles than medial LMC neurons. Why then do we see such comparatively subtle differences between individual medial and lateral LMC neurons? The most likely explanation seems to be the recording method; rather than using a suction pipette to record the summed activity of an entire nerve root, we are examining the firing of individual neurons within the lateral motor column. The differences in activity between nerve roots seen by Milner et al. [4] may be a result of temporal differences between the initiation of firing across a motor pool that, when combined with the subtle differences we see in cycle duration between medial and lateral LMC neurons (Fig. 2G), and the relatively short distance interactions upon which activity propagation depends at early stages (Fig. 7G) results in more obvious differences between motor pools at the nerve root level of resolution. It is difficult to compare the activity of single motor neurons in this study to that seen in older embryos [65], [66]. At later stages, motor neuron episodic activity consists of one synchronous depolarisation in both flexors and extensors, which are likely to be the cycles detected in our recordings, followed by alternating patterns of complex episodes consisting of many cycles, and demonstrating a considerable maturation of the motor circuit between HH St. 30 and HH St. 36.

### Pharmacological Treatments Reveal Influences of Neurotransmitter Action

Our probing of the motor circuit using nicotinic, muscarinic and GABA-ergic antagonists is the first such study to analyze this circuit at the level of individual neurons by use of both patch-clamp and calcium imaging at this early stage of development. This analysis shows that that acute blockade of nicotinic stimulation silences the entire motor circuit, whereas muscarinic stimulation has a more modulatory role. It has been demonstrated previously that motor circuit activity returns after a period of sustained application of DTC [4], however this phenomena was not assessed in this study due to the length of our recordings. As seen in Borda et al. [67], muscarinic responses can be biphasic, with low levels of inhibition by scopolamine resulting in increased cycle frequency, but high levels of inhibition resulting in a decrease in frequency. Application of cholinergic agonists during single-cell recording suggests important differences between the different neurotransmitter systems operating in the early motor circuit. ACh application disrupts episodes, though there is a large increase in cycle frequency; conversely nicotine stimulation has relatively minor effects upon firing, though episodic activity did not return after cessation of nicotine application, suggesting that motor neurons had become desensitized to nicotine and now required the exogenous application of the drug to remain active. Given that nicotinic signaling is absolutely required for circuit firing (Fig. 3A), and that circuit firing can persist in the absence of muscarinic signaling (Fig. 5D and E, Tables 3 & 4), these data suggest that nicotinic stimulation is critical for cycle initiation, whereas muscarinic stimulation allows potentials initiated by nicotinic stimulation to reach the level required for full cycle firing. Indeed, these proposed roles fit with the metabotropic and ionotropic actions of muscarinic and nicotinic receptors, respectively.

GABA-A inhibition with PTX destabilized circuit firing (Fig. 3E), resulting in a reduction in the frequency of episodes, with neurons failing to fire full ‘cycles’, but rather firing in sequences of smaller depolarisations (Fig. 3I) that may reflect repeated failure to reach the threshold at which the full complement of channels required for cycle firing are open. Unlike cholinergic stimulation, GABA application does not disrupt episodic activity, but does increase its frequency (Fig. 3F), alongside an increase in the baseline ‘noise’ of the recorded neurons. These results suggest that at least until HH St. 30, GABA is critical for regulating the ‘tone’ of the motor circuit, priming it for firing; in the absence of GABA-A signaling, fewer depolarisations reach threshold, and while this results in a decrease in cycle and episode frequency it also allows the firing of more sub-threshold cycles. Calcium imaging reveals similar results, with a reduction in the frequency of episodes during PTX application; the increased number of sub-threshold cycles seen in single-cell patch-clamp recordings cannot be seen in calcium imaging, as such cycles do not initiate an increase in calcium indicator intensity, as seen in Fig. 4C.

The application of serotonin to the spinal cord increases the frequency of episodes; given that there are no SERT1^+^ neurons in the spinal cord at this age (Table 1), our data suggests that descending serotonergic inputs are active in regulating the frequency of motor neuron firing from early stages. Additionally, in every case where drugs added to the spinal cord increase the frequency of episodic activity, we see a decrease in the number of cycles per episode (Fig. 3B, D, F, H), suggesting the presence of post-episodic depression in the spinal cord at very early stages of activity development [68].

Together with our data examining differential expression of neurotransmitter receptor mRNAs across the motor circuit, these data imply the existence of a system in which small initial differences in neurotransmitter receptor complement between neuronal subtypes result in different intracellular responses to undifferentiated activity patterns, influencing the connectivity of neurons in the motor circuit. These differences in connectivity may then be reinforced by not only the initial strength of a connection, but also the transmitters active at that connection and the different influences of these transmitter types upon activity at a given synapse, leading to the differentiation of activity patterns later in circuit development. If such a mechanism is occurring, inhibiting the activity of specific neurons within this circuit with Kir2.1 [12], should lead to alteration of their dendritic trees. An alternative method to probe this mechanism would be to increase the homogeneity of the motor circuit by genetic ablation of specific neurotransmitter receptor genes, which should lead to similar dendritic patterning of motor pools normally differentiated by expression of the ablated neurotransmitter receptor.

### Emergence of Synchronicity

Whilst electrical activity has repeatedly been demonstrated for emerging neuronal circuits [1], [2], [6], [69], [70], the very earliest patterns of activity in the spinal cord have only been reported for zebrafish [70], in which random firing of neurons becomes synchronous over time. The data presented here imply that this phenomenon might occur in the chick spinal cord between HH St. 23 and HH St. 30. The emergence of this patterned activity could plausibly be a result of the appearance of specific neurotransmitter receptor subunits (e.g. *CHRNA7*). However, if the mechanism were as such, it would be expected that application of antagonists to these receptors would have an immediate effect upon the synchrony of motor neuron firing, which is not the case; none of the antagonists examined in this study were able to produce a decrease in the synchrony of the system, and only DTC was capable of entirely silencing the circuit. Given the recurrent projections proposed in the motor circuit [27], [49], [50], and that all neurotransmitters assayed in this study and others [4], [27] are excitatory at this stage, it seems likely that recurrent excitation underlies the emergence of synchronicity. Our results do not preclude a role for electrical coupling of spinal neurons in the chick, as previously demonstrated by Milner et al., [4], though blockade of spontaneous activity with gap junction inhibitors only occurred after a long time course in said study (∼50 minutes), suggesting that the motor circuit is resistant to such perturbations. Whilst glial cells are known to propagate calcium waves [71] and may possibly contribute to the emergence of synchronicity reported here, the time course of their wave propagation is slow (∼20–30 μm/s) and hence difficult to reconcile with the observed near instant synchrony of LMC neuron activity.

In a model of recurrent excitatory chemical synapses, initially small clusters of active neurons recruit and entrain their neighbours to fire with them; an effect which may be enhanced by electrical coupling, and potentiated by glial waves. The size of these synchronized neighbourhoods would increase exponentially, the precise dynamics of recruitment being determined by the level of connectivity and recruitment power of motor neurons. Indeed, in experiments performed by Warp et al. [70], inactivation of neurons in the Zebrafish spinal cord with light-induced halorhodopsin currents lead to a reduction in the correlated firing of spinal neurons, as predicted by such a model. Depending on the strength and degree of connectivity within the circuit, the emergence of synchronicity may be more or less robust to perturbations. One might expect, for example, that the synchronicity of a network of neurons that only connect to one or two others, even very strongly, would be susceptible to silencing of small numbers of neurons. Conversely, the synchronicity of a network with many weak links between neurons may be more robust to such silencing; even though the links between neurons are weaker, their cumulative weight would be sufficient to allow the emergence of synchrony when small portions of the network were silenced.

### Conclusions

Together, our data describe the earliest stages of the motor circuit in terms of transmitter diversity, pharmacology and activity patterns, and imply that whilst activity patterns within the circuit at this stage are relatively simple, the circuitry that underlies these patterns is highly complex, likely setting the stage for the later differentiation of activity patterns prior to the onset of sensory experience.

## Materials and Methods

### Animals

Fertilized chick eggs (Couvoir Simetin, Mirabel, QC, Canada) were stored for a maximum of one week at 18°C, then incubated at 38°C and staged according to standard protocols [72]. Embryos were sacrificed by means of decapitation using forceps which conforms to Canadian Council On Animal Care regulations.

### *In ovo* Electroporation

Chicken spinal cord electroporation of expression plasmids was performed at Hamburger-Hamilton (HH) stages (St.) 18/19 [72], generally as described [73]. In brief, a solution of plasmid DNA in dH_2_0 was injected into the caudal half of the neural tube through a small eggshell window under a Discovery V12 stereomicroscope (Zeiss). Current produced by the TSS20 Ovodyne electroporator (Intracel; settings: 30 V, 5 pulses 50 ms wide in a 1 s interval) was applied to the prospective lumbar region of the neural tube with platinum/iridium electrodes (FHC). Shell windows were sealed with Parafilm (Pechiney Plastic Packaging Company) and incubated at 37°C until harvesting at HH St. 30 (E6). The efficiency of electroporation varied between 5 and 30% of all LMC neurons depending on the construct and DNA concentration used.

### *In situ* Hybridization and Immunohistochemistry

cDNA probes were designed against neurotransmitter transporters, synthetic enzymes and receptors listed in Table 1, by using the primer3 primer designer (http://primer3.ut.ee/) and mRNA sequences from the NIH nucleotide database (http://www.ncbi.nlm.nih.gov/nuccore/). Primer pairs were selected based upon subsequent chicken nucleotide BLAST (http://blast.ncbi.nlm.nih.gov/Blast.cgi) searches of the probe sequence; primer pairs were only selected if the corresponding probe specifically identified the target mRNA in database searches. For probes directed against mRNAs that undergo alternative splicing, a sequence common to all isoforms was chosen as the target for probe design. The T7 RNA polymerase recognition sequence was added to the 5′ end of the reverse probe. Probe sequences are available upon request. Digoxygenin-labeled riboprobes were synthesized by using the amplified fragments as templates, following standard protocols [74]. Embryos were allowed to develop until the indicated stages, then fixed in 4% paraformaldehyde, followed by cryopreservation in 30% sucrose in PBS, embedding in OCT (Tissue-Tek) and cryosectioning at 12 μm thickness. In situ hybridizations were then carried out according to standard protocols [74], using a BCiP/NBT/Levamisole development solution. Immunohistochemistry on adjacent sections was carried out using primary antibodies directed against Isl1 (3F7, Developmental Studies Hybridoma Bank (DSHB), Iowa), NKCC1 (T4, DSHB, Iowa) or KCC2 (NeuroMab, UC Davis), followed by appropriate secondary antibodies conjugated to fluorochromes (Life Technologies, USA). For anti-NKCC1 antiserum use, antigen retrieval consisting of 5 minutes immersion in 1% SDS was performed prior to immunostaining. Images were captured on a Leica DM6000 microscope using Volocity (Improvision) software, or a Zeiss LSM 710 confocal microscope using Zen (Zeiss) software. Images of *GAD1 in situs* were treated with the ‘Remove Outliers’ filter in FIJI (ImageJ, NIH) to remove speckles caused by bubbles in the mounting media.

### Calcium Imaging

Chicken embryos at HH St. 18–19 were co-electroporated with *Hb9-eGFP* (5 μg/μL) and *R-GECO* (1 μg/μL; [47] expression plasmids as above, then harvested at either HH St. 26 or HH St. 30. For experiments examining the emergence of synchronized activity, embryos were harvested at the specified age into a bath of circulating, oxygenated Tyrode’s solution held at 29°C. A bolus of fluo-4 AM dissolved in DMSO was injected into the limb, and the embryos were incubated for 90 minutes to allow uptake of the dye into motor neurons. Spinal cords (including lower cervical through to sacral levels) were dissected and placed into an imaging chamber with flowing oxygenated Tyrode’s solution (2 mL/min; 140 mM NaCl, 3 mM KCl, 17 mM NaHCO_3_, 12 mM glucose, 2 mM CaCl_2_, 1 mM MgCl_2_; Fisher Scientific) held at 30°C. Spinal cords were imaged at 1 Hz for 10 minutes using either a Zeiss Axio Observer Z1 microscope with a CSU-X1M dual cam 5000 spinning disk (Fig. 5), or an Olympus BX-51 WI epifluorescence microscope with a water immersion lens (Fig. 6–7). After 10 minutes of imaging of baseline activity, drugs dissolved in Tyrode’s solution were added at the indicated concentrations for 20 minutes by means of a syringe pump (Harvard Apparatus) injecting 10 mM stock solutions of drug in-line with the flow of Tyrode’s solution. Differences exist in the shape, volume and perfusion characteristics between the chambers used for electrophysiological recording and calcium imaging; the electrophysiological chamber has a small volume (0.75 ml), which is rapidly replaced by removing liquid from the base of the recording chamber. In contrast, the volume of the circular calcium imaging chamber is larger (3.75 ml), and the fluid flow is removed from the top of the bath, resulting in slower fluid replacement. In calcium imaging experiments DTC was used at a higher concentration (10 μM vs 5 μM) to silence neuronal activity. Imaging was then performed again after washout of the drug to verify the return of activity. Calcium activity was assessed using ImageJ (NIH) and a custom MATLAB (Mathworks) algorithm which detects large changes (>2 standard deviations from the mean differential) in calcium indicator fluorescence intensity (Fig. 4B), defined here as ‘cycles’. Cycles were classified as synchronous if they occurred in >60% of the labelled neurons in a field.

### Electrophysiology

Embryos electroporated with *Hb9-EGFP* (Fig. 2) were allowed to develop until HH St. 30, then spinal cords were dissected, and placed ventral side up in a recording chamber with oxygenated Tyrode’s solution flowing at 2 mL/min. Neurons were patched based upon either location within the LMC (Fig. 2,4), or LMC location in conjunction with *Hb9-eGFP* expression (Fig. 3). The standard pipette solution contained (in mM): 130 KCl, 0.2 CaCl_2_, 2 MgCl_2_, 1 EGTA, 10 HEPES, 2 Na_2_ATP 0.5 Na_2_GTP, 1 Na_2_ Phosphocreatine. For experiments in Fig. 2, 10 μM Alexafluo 568 (Life Technologies) was added to the pipette solution to label recorded neurons. Patch-clamp recordings were obtained from the soma of motor neurons using a HEKA EPC10 amplifier; voltage was filtered using a bandpass filter (0.3 Hz–2 kHz), and sampled at 1 kHz, using patch pipettes (2–8 MΩ) pulled from thick-walled borosilicate glass capillaries (1.50 mm outer diameter, 0.64 mm wall thickness, Harvard Apparatus, USA). For Isl1^+^ vs. Lim1^+^ motor neuron comparisons, spinal cords were immunostained with antibodies directed against Foxp1 (a kind gift from Bennett Novitch, UCLA), and either Isl1 or Lim1 (DSHB, Iowa). For experiments in Fig. 3, drugs were added at the indicated concentrations by means of a syringe pump (KD Scientific) injecting 10 mM stock solutions of drug in-line with the flow of Tyrode’s solution; at least 2 embryos for each condition were recorded from, with repeatable results.

## References

[1] Garaschuk O, Hanse E, Konnerth A (1998) Developmental profile and synaptic origin of early network oscillations in the CA1 region of rat neonatal hippocampus. The Journal of physiology 507 (Pt 1): 219–236. 10.1111/j.1469-7793.1998.219bu.x PMC2230780 9490842

[2] Garaschuk O, Linn J, Eilers J, Konnerth A (2000) Large-scale oscillatory calcium waves in the immature cortex. Nature neuroscience 3: 452–459. 10769384 10.1038/74823

[3] Hamburger V, Balaban M (1963) Observations and experiments on spontaneous rhythmical behavior in the chick embryo. Developmental biology 6: 533–545. 13952299 10.1016/0012-1606(63)90140-4

[4] Milner LD, Landmesser LT (1999) Cholinergic and GABAergic inputs drive patterned spontaneous motoneuron activity before target contact. J Neurosci 19: 3007–3022. 10191318 10.1523/JNEUROSCI.19-08-03007.1999 PMC6782286

[5] Mire E, Mezzera C, Leyva-Diaz E, Paternain AV, Squarzoni P, et al (2013) Spontaneous activity regulates Robo1 transcription to mediate a switch in thalamocortical axon growth. Nature neuroscience 15: 1134–1143. 10.1038/nn.3160 22772332

[6] Watt AJ, Cuntz H, Mori M, Nusser Z, Sjostrom PJ, et al (2009) Traveling waves in developing cerebellar cortex mediated by asymmetrical Purkinje cell connectivity. Nature neuroscience 12: 463–473. 19287389 10.1038/nn.2285 PMC2912499

[7] Borodinsky LN, Root CM, Cronin JA, Sann SB, Gu X, et al (2004) Activity-dependent homeostatic specification of transmitter expression in embryonic neurons. Nature 429: 523–530. 15175743 10.1038/nature02518

[8] Demarque M, Spitzer NC (2010) Activity-dependent expression of Lmx1b regulates specification of serotonergic neurons modulating swimming behavior. Neuron 67: 321–334. 20670838 10.1016/j.neuron.2010.06.006 PMC2913149

[9] Plazas PV, Nicol X, Spitzer NC (2013) Activity-dependent competition regulates motor neuron axon pathfinding via PlexinA3. Proceedings of the National Academy of Sciences of the United States of America 110: 1524–1529. 23302694 10.1073/pnas.1213048110 PMC3557035

[10] Hanson MG, Landmesser LT (2004) Normal patterns of spontaneous activity are required for correct motor axon guidance and the expression of specific guidance molecules. Neuron 43: 687–701. 15339650 10.1016/j.neuron.2004.08.018

[11] Nicol X, Voyatzis S, Muzerelle A, Narboux-Neme N, Sudhof TC, et al (2007) cAMP oscillations and retinal activity are permissive for ephrin signaling during the establishment of the retinotopic map. Nature neuroscience 10: 340–347. 17259982 10.1038/nn1842

[12] Benjumeda I, Escalante A, Law C, Morales D, Chauvin G, et al (2013) Uncoupling of EphA/ephrinA Signaling and Spontaneous Activity in Neural Circuit Wiring. J Neurosci 33: 18208–18218. 24227729 10.1523/JNEUROSCI.1931-13.2013 PMC3831575

[13] Hubel DH, Wiesel TN, LeVay S (1977) Plasticity of ocular dominance columns in monkey striate cortex. Philosophical transactions of the Royal Society of London 278: 377–409. 19791 10.1098/rstb.1977.0050

[14] Ni X, Martin-Caraballo M (2010) Differential effect of glutamate receptor blockade on dendritic outgrowth in chicken lumbar motoneurons. Neuropharmacology 58: 593–604. 19995566 10.1016/j.neuropharm.2009.11.016

[15] Vogel MW, Prittie J (1995) Purkinje cell dendritic arbors in chick embryos following chronic treatment with an N-methyl-D-aspartate receptor antagonist. Journal of neurobiology 26: 537–552. 7602317 10.1002/neu.480260407

[16] Briscoe J, Pierani A, Jessell TM, Ericson J (2000) A homeodomain protein code specifies progenitor cell identity and neuronal fate in the ventral neural tube. Cell 101: 435–445. 10830170 10.1016/s0092-8674(00)80853-3

[17] Dalla Torre di Sanguinetto SA, Dasen JS, Arber S (2008) Transcriptional mechanisms controlling motor neuron diversity and connectivity. Current opinion in neurobiology 18: 36–43. 18524570 10.1016/j.conb.2008.04.002

[18] Ericson J, Briscoe J, Rashbass P, van Heyningen V, Jessell TM (1997) Graded sonic hedgehog signaling and the specification of cell fate in the ventral neural tube. Cold Spring Harbor symposia on quantitative biology 62: 451–466. 9598380

[19] Sockanathan S, Jessell TM (1998) Motor neuron-derived retinoid signaling specifies the subtype identity of spinal motor neurons. Cell 94: 503–514. 9727493 10.1016/s0092-8674(00)81591-3

[20] Tsuchida T, Ensini M, Morton SB, Baldassare M, Edlund T, et al (1994) Topographic organization of embryonic motor neurons defined by expression of LIM homeobox genes. Cell 79: 957–970. 7528105 10.1016/0092-8674(94)90027-2

[21] O’Donovan MJ, Landmesser L (1987) The development of hindlimb motor activity studied in the isolated spinal cord of the chick embryo. J Neurosci 7: 3256–3264. 3668626 10.1523/JNEUROSCI.07-10-03256.1987 PMC6569173

[22] Provine RR, Sharma SC, Sandel TT, Hamburger V (1970) Electrical activity in the spinal cord of the chick embryo, in situ. Proceedings of the National Academy of Sciences of the United States of America 65: 508–515. 5267135 10.1073/pnas.65.3.508 PMC282936

[23] Chub N, O’Donovan MJ (1998) Blockade and recovery of spontaneous rhythmic activity after application of neurotransmitter antagonists to spinal networks of the chick embryo. J Neurosci 18: 294–306. 9412508 10.1523/JNEUROSCI.18-01-00294.1998 PMC6793395

[24] Mendelson B, Koerber HR, Frank E (1992) Development of cutaneous and proprioceptive afferent projections in the chick spinal cord. Neuroscience letters 138: 72–76. 1383880 10.1016/0304-3940(92)90475-m

[25] O’Donovan M, Ho S, Yee W (1994) Calcium imaging of rhythmic network activity in the developing spinal cord of the chick embryo. J Neurosci 14: 6354–6369. 7965041 10.1523/JNEUROSCI.14-11-06354.1994 PMC6577302

[26] Wang S, Polo-Parada L, Landmesser LT (2009) Characterization of rhythmic Ca2+ transients in early embryonic chick motoneurons: Ca2+ sources and effects of altered activation of transmitter receptors. J Neurosci 29: 15232–15244. 19955376 10.1523/JNEUROSCI.3809-09.2009 PMC2956416

[27] Hanson MG, Landmesser LT (2003) Characterization of the circuits that generate spontaneous episodes of activity in the early embryonic mouse spinal cord. J Neurosci 23: 587–600. 12533619 10.1523/JNEUROSCI.23-02-00587.2003 PMC6741864

[28] O’Donovan MJ (1989) Motor activity in the isolated spinal cord of the chick embryo: synaptic drive and firing pattern of single motoneurons. J Neurosci 9: 943–958. 2926486 10.1523/JNEUROSCI.09-03-00943.1989 PMC6569958

[29] Sernagor E, Chub N, Ritter A, O’Donovan MJ (1995) Pharmacological characterization of the rhythmic synaptic drive onto lumbosacral motoneurons in the chick embryo spinal cord. J Neurosci 15: 7452–7464. 7472497 10.1523/JNEUROSCI.15-11-07452.1995 PMC6578097

[30] Kastanenka KV, Landmesser LT (2010) In vivo activation of channelrhodopsin-2 reveals that normal patterns of spontaneous activity are required for motoneuron guidance and maintenance of guidance molecules. J Neurosci 30: 10575–10585. 20686000 10.1523/JNEUROSCI.2773-10.2010 PMC2934783

[31] Landmesser LT, Szente M (1986) Activation patterns of embryonic chick hind-limb muscles following blockade of activity and motoneurone cell death. The Journal of physiology 380: 157–174. 3612563 10.1113/jphysiol.1986.sp016278 PMC1182930

[32] Myers CP, Lewcock JW, Hanson MG, Gosgnach S, Aimone JB, et al (2005) Cholinergic input is required during embryonic development to mediate proper assembly of spinal locomotor circuits. Neuron 46: 37–49. 15820692 10.1016/j.neuron.2005.02.022

[33] Temkin R, Lowe D, Jensen P, Hatt H, Smith DO (1997) Expression of glutamate receptor subunits in alpha-motoneurons. Brain Res Mol Brain Res 52: 38–45. 9450675 10.1016/s0169-328x(97)00249-0

[34] Hollyday M, Hamburger V (1977) An autoradiographic study of the formation of the lateral motor column in the chick embryo. Brain research 132: 197–208. 890480 10.1016/0006-8993(77)90416-4

[35] Tosney KW, Landmesser LT (1985) Development of the major pathways for neurite outgrowth in the chick hindlimb. Developmental biology 109: 193–214. 2985457 10.1016/0012-1606(85)90360-4

[36] Landmesser L, Morris DG (1975) The development of functional innervation in the hind limb of the chick embryo. The Journal of physiology 249: 301–326. 1177095 10.1113/jphysiol.1975.sp011017 PMC1309576

[37] Landmesser L (1978) The distribution of motoneurones supplying chick hind limb muscles. The Journal of physiology 284: 371–389. 731549 10.1113/jphysiol.1978.sp012545 PMC1282826

[38] Rivera C, Voipio J, Payne JA, Ruusuvuori E, Lahtinen H, et al (1999) The K+/Cl- co-transporter KCC2 renders GABA hyperpolarizing during neuronal maturation. Nature 397: 251–255. 9930699 10.1038/16697

[39] Landmesser LT, O’Donovan MJ (1984) Activation patterns of embryonic chick hind limb muscles recorded in ovo and in an isolated spinal cord preparation. The Journal of physiology 347: 189–204. 6707956 10.1113/jphysiol.1984.sp015061 PMC1199442

[40] Gerzanich V, Wang F, Kuryatov A, Lindstrom J (1998) alpha 5 Subunit alters desensitization, pharmacology, Ca++ permeability and Ca++ modulation of human neuronal alpha 3 nicotinic receptors. The Journal of pharmacology and experimental therapeutics 286: 311–320. 9655874

[41] Wang F, Gerzanich V, Wells GB, Anand R, Peng X, et al (1996) Assembly of human neuronal nicotinic receptor alpha5 subunits with alpha3, beta2, and beta4 subunits. The Journal of biological chemistry 271: 17656–17665. 8663494 10.1074/jbc.271.30.17656

[42] Holley JA, Wimer CC, Vaughn JE (1982) Quantitative analyses of neuronal development in the lateral motor column of mouse spinal cord. I. Genetically associated variations in somal growth patterns. The Journal of comparative neurology 207: 314–321. 7119144 10.1002/cne.902070403

[43] Koch C (1999) Biophysics of Computation. New York: Oxford University Press.

[44] Arber S, Han B, Mendelsohn M, Smith M, Jessell TM, et al (1999) Requirement for the homeobox gene Hb9 in the consolidation of motor neuron identity. Neuron 23: 659–674. 10482234 10.1016/s0896-6273(01)80026-x

[45] Cazalets JR, Sqalli-Houssaini Y, Clarac F (1992) Activation of the central pattern generators for locomotion by serotonin and excitatory amino acids in neonatal rat. The Journal of physiology 455: 187–204. 1362441 10.1113/jphysiol.1992.sp019296 PMC1175639

[46] Cowley KC, Schmidt BJ (1994) A comparison of motor patterns induced by N-methyl-D-aspartate, acetylcholine and serotonin in the in vitro neonatal rat spinal cord. Neuroscience letters 171: 147–150. 8084477 10.1016/0304-3940(94)90626-2

[47] Zhao Y, Araki S, Wu J, Teramoto T, Chang YF, et al (2011) An expanded palette of genetically encoded Ca(2)(+) indicators. Science (New York, NY 333: 1888–1891. 10.1126/science.1208592 PMC3560286 21903779

[48] Tsien RY (1981) A non-disruptive technique for loading calcium buffers and indicators into cells. Nature 290: 527–528. 7219539 10.1038/290527a0

[49] Cullheim S, Kellerth JO, Conradi S (1977) Evidence for direct synaptic interconnections between cat spinal alpha-motoneurons via the recurrent axon collaterals: a morphological study using intracellular injection of horseradish peroxidase. Brain research 132: 1–10. 70258 10.1016/0006-8993(77)90702-8

[50] Perrins R, Roberts A (1995) Cholinergic and electrical motoneuron-to-motoneuron synapses contribute to on-cycle excitation during swimming in Xenopus embryos. Journal of neurophysiology 73: 1005–1012. 7608750 10.1152/jn.1995.73.3.1005

[51] Kuryatov A, Onksen J, Lindstrom J (2008) Roles of accessory subunits in alpha4beta2(*) nicotinic receptors. Molecular pharmacology 74: 132–143. 18381563 10.1124/mol.108.046789

[52] Lozada AF, Wang X, Gounko NV, Massey KA, Duan J, et al (2012) Glutamatergic synapse formation is promoted by alpha7-containing nicotinic acetylcholine receptors. J Neurosci 32: 7651–7661. 22649244 10.1523/JNEUROSCI.6246-11.2012 PMC3370670

[53] Liu Z, Neff RA, Berg DK (2006) Sequential interplay of nicotinic and GABAergic signaling guides neuronal development. Science (New York, NY 314: 1610–1613. 10.1126/science.1134246 17158331

[54] Karim N, Wellendorph P, Absalom N, Johnston GA, Hanrahan JR, et al (2013) Potency of GABA at human recombinant GABA(A) receptors expressed in Xenopus oocytes: a mini review. Amino acids 44: 1139–1149. 23385381 10.1007/s00726-012-1456-y

[55] Cline H, Haas K (2008) The regulation of dendritic arbor development and plasticity by glutamatergic synaptic input: a review of the synaptotrophic hypothesis. The Journal of physiology 586: 1509–1517. 18202093 10.1113/jphysiol.2007.150029 PMC2375708

[56] Dhande OS, Bhatt S, Anishchenko A, Elstrott J, Iwasato T, et al (2012) Role of adenylate cyclase 1 in retinofugal map development. The Journal of comparative neurology 520: 1562–1583. 22102330 10.1002/cne.23000 PMC3563095

[57] Jablonski AM, Kalb RG (2013) GluA1 promotes the activity-dependent development of motor circuitry in the developing segmental spinal cord. Annals of the New York Academy of Sciences 1279: 54–59. 23531002 10.1111/nyas.12053 PMC4205102

[58] Ravary A, Muzerelle A, Herve D, Pascoli V, Ba-Charvet KN, et al (2003) Adenylate cyclase 1 as a key actor in the refinement of retinal projection maps. J Neurosci 23: 2228–2238. 12657682 10.1523/JNEUROSCI.23-06-02228.2003 PMC6742000

[59] Vrieseling E, Arber S (2006) Target-induced transcriptional control of dendritic patterning and connectivity in motor neurons by the ETS gene Pea3. Cell 127: 1439–1452. 17190606 10.1016/j.cell.2006.10.042

[60] Mendez J, Kadia TM, Somayazula RK, El-Badawi KI, Cowen DS (1999) Differential coupling of serotonin 5-HT1A and 5-HT1B receptors to activation of ERK2 and inhibition of adenylyl cyclase in transfected CHO cells. Journal of neurochemistry 73: 162–168. 10386967 10.1046/j.1471-4159.1999.0730162.x

[61] Briz V, Hsu YT, Li Y, Lee E, Bi X, et al (2013) Calpain-2-mediated PTEN degradation contributes to BDNF-induced stimulation of dendritic protein synthesis. J Neurosci 33: 4317–4328. 23467348 10.1523/JNEUROSCI.4907-12.2013 PMC3657575

[62] Lein P, Guo X, Shi G, Moholt-Siebert M, Bruun D, et al (2007) The novel GTPase Rit differentially regulates axonal and dendritic growth. J Neurosci 27: 4725–4736. 17460085 10.1523/JNEUROSCI.5633-06.2007 PMC3495986

[63] Wolfram V, Southall TD, Brand AH, Baines RA (2012) The LIM-homeodomain protein islet dictates motor neuron electrical properties by regulating K(+) channel expression. Neuron 75: 663–674. 22920257 10.1016/j.neuron.2012.06.015 PMC3427859

[64] Root CM, Velázquez-Ulloa NA, Monsalve GC, Minkova E, Spitzer NC (2008) Embryoniclly expressed GABA and glutamate drive electrical activity rgulating neurotransmitter specification. J Neurosci 28: 4777–4784. 18448654 10.1523/JNEUROSCI.4873-07.2008 PMC3318922

[65] O’Donovan M, Sernagor E, Sholomenko G, Ho S, Antal M, et al (1992) Development of spinal motor networks in the chick embryo. The Journal of experimental zoology 261: 261–273. 1629659 10.1002/jez.1402610306

[66] Sernagor E, O’Donovan MJ (1991) Whole-cell patch clamp recordings from rhythmically active motoneurons in the isolated spinal cord of the chick embryo. Neuroscience letters 128: 211–216. 1945040 10.1016/0304-3940(91)90263-s

[67] Borda T, Genaro A, Sterin-Borda L, Cremaschi G (1998) Involvement of endogenous nitric oxide signalling system in brain muscarinic acetylcholine receptor activation. J Neural Transm 105: 193–204. 9660097 10.1007/s007020050048

[68] Fedirchuk B, Wenner P, Whelan PJ, Ho S, Tabak J, et al (1999) Spontaneous network activity transiently depresses synaptic transmission in the embryonic chick spinal cord. J Neurosci 19: 2102–2112. 10066263 10.1523/JNEUROSCI.19-06-02102.1999 PMC6782567

[69] Tritsch NX, Yi E, Gale JE, Glowatzki E, Bergles DE (2007) The origin of spontaneous activity in the developing auditory system. Nature 450: 50–55. 17972875 10.1038/nature06233

[70] Warp E, Agarwal G, Wyart C, Friedmann D, Oldfield CS, et al (2011) Emergence of patterned activity in the developing zebrafish spinal cord. Curr Biol 22: 93–102. 22197243 10.1016/j.cub.2011.12.002 PMC3267884

[71] Newman EA (2001) Propagation of intercellular calcium waves in retinal astrocytes and Müller cells. J Neurosci 21: 2215–2223. 11264297 10.1523/JNEUROSCI.21-07-02215.2001 PMC2409971

[72] Hamburger V, Hamilton HL (1951) A series of normal stages in the development of the chick embryo. Journal of Morphology 88: 49–92. 24539719

[73] Croteau LP, Kania A (2011) Optimisation of in ovo electroporation of the chick neural tube. Journal of neuroscience methods 201: 381–384. 21871488 10.1016/j.jneumeth.2011.08.012

[74] Kao TJ, Palmesino E, Kania A (2009) SRC family kinases are required for limb trajectory selection by spinal motor axons. J Neurosci 29: 5690–5700. 19403835 10.1523/JNEUROSCI.0265-09.2009 PMC6665840

